# Disruption of pulmonary microvascular endothelial barrier by dysregulated claudin-8 and claudin-4: uncovered mechanisms in porcine reproductive and respiratory syndrome virus infection

**DOI:** 10.1007/s00018-024-05282-4

**Published:** 2024-05-28

**Authors:** Weifeng Sun, Weixin Wu, Xinyu Fang, Xinna Ge, Yongning Zhang, Jun Han, Xin Guo, Lei Zhou, Hanchun Yang

**Affiliations:** 1 National Key Laboratory of Veterinary Public Health Safety, College of Veterinary Medicine, China Agricultural University, Beijing, 100193 People’s Republic of China; 2 Key Laboratory of Animal Epidemiology of Ministry of Agriculture and Rural Affairs, College of Veterinary Medicine, China Agricultural University, Beijing, 100193 People’s Republic of China; 3 China Institute of Veterinary Drug Control, Beijing, 100081 People’s Republic of China

**Keywords:** Acute lung injury (ALI), Pulmonary microvascular endothelial barrier, Claudin-4 (CLDN4), Claudin-8 (CLDN8), *miR-185*, Porcine reproductive and respiratory syndrome virus (PRRSV)

## Abstract

The pulmonary endothelium is a dynamic and metabolically active monolayer of endothelial cells. Dysfunction of the pulmonary endothelial barrier plays a crucial role in the acute lung injury (ALI) and acute respiratory distress syndrome (ARDS), frequently observed in the context of viral pneumonia. Dysregulation of tight junction proteins can lead to the disruption of the endothelial barrier and subsequent leakage. Here, the highly pathogenic porcine reproductive and respiratory syndrome virus (HP-PRRSV) served as an ideal model for studying ALI and ARDS. The alveolar lavage fluid of pigs infected with HP-PRRSV, and the supernatant of HP-PRRSV infected pulmonary alveolar macrophages were respectively collected to treat the pulmonary microvascular endothelial cells (PMVECs) in Transwell culture system to explore the mechanism of pulmonary microvascular endothelial barrier leakage caused by viral infection. Cytokine screening, addition and blocking experiments revealed that proinflammatory cytokines IL-1β and TNF-α, secreted by HP-PRRSV-infected macrophages, disrupt the pulmonary microvascular endothelial barrier by downregulating claudin-8 and upregulating claudin-4 synergistically. Additionally, three transcription factors interleukin enhancer binding factor 2 (ILF2), general transcription factor III C subunit 2 (GTF3C2), and thyroid hormone receptor-associated protein 3 (THRAP3), were identified to accumulate in the nucleus of PMVECs, regulating the transcription of claudin-8 and claudin-4. Meanwhile, the upregulation of *ssc-miR-185* was found to suppress claudin-8 expression via post-transcriptional inhibition. This study not only reveals the molecular mechanisms by which HP-PRRSV infection causes endothelial barrier leakage in acute lung injury, but also provides novel insights into the function and regulation of tight junctions in vascular homeostasis.

**Supplementary Information:**

The online version contains supplementary material available at 10.1007/s00018-024-05282-4.

## Introduction

Acute lung injury (ALI) is a key pathophysiological process contributing to lung dysfunction [1], often observed during pneumonia caused by viral pathogens, such as severe acute respiratory syndrome coronavirus-2 (SARS-CoV-2) or highly pathogenic avian influenza (HPAI) virus for human, as well as porcine reproductive and respiratory syndrome virus (PRRSV) for pig [2–4]. Porcine reproductive and respiratory syndrome virus (PRRSV), which causes reproductive failure in pregnant sows and respiratory disorders in pigs of all ages, is one of the most economically significant viral pathogens affecting the global pork industry [5]. Highly pathogenic PRRSV (HP-PRRSV) infection is known to induce severe pneumonia, characterized by the destruction of lung structure, extensive hemorrhage, and significant infiltration of inflammatory cells. This makes it an ideal model for studying the pathological processes of acute lung injury (ALI) [4]. Recent transcriptome analysis of pulmonary microvascular endothelial cells (PMVECs) co-cultured with HP-PRRSV-infected pulmonary alveolar macrophages (PAMs) has shown that several tight junction (TJ) proteins were dysregulated at both the mRNA and protein levels. However, the specific functional TJs responsible for endothelial barrier permeability remain to be elucidated [6].

The vascular endothelium, lining the inner surface of blood vessels, maintains an intact barrier to prevent the leakage of circulating solutes, plasma proteins, liquid, and immune cells out of the blood vessel [7]. It serves as the first interface for circulating blood components to interact with cells of the vascular wall and surrounding extravascular tissues. Intercellular junctions between adjacent PMVECs, including gap junctions (GJs), adherens junctions (AJs), and tight junctions (TJs), are crucial for maintaining the integrity of the endothelial barrier [8, 9]. Among these, TJs seal the intercellular space, which is vital for maintaining vascular homeostasis. AJs form strong mechanical connections among neighboring cells, while GJs primarily facilitate intercellular communication [10]. TJs are dynamic structures that can change under both physiological and pathological conditions, and their dysfunction can disrupt the endothelial barrier, affecting vascular homeostasis and leading to the leakage of fluid, macromolecules, and even cells [11].

Claudins (CLDNs) are a group of four-transmembrane-spanning TJ proteins with molecular weights ranging from 20 to 27 kDa. In mammals, there are 27 members of this family, which are categorized into barrier-forming and channel-/pore-forming proteins. They form a complex network that controls the intercellular permeability of both epithelial and endothelial tissues [12, 13]. Due to their tissue-specific expression and complex interaction patterns, different claudins may play distinct roles in regulating fluid diffusion between neighboring cells, known as paracellular flux. For instance, claudin 4 (CLDN4) has been reported to form a paracellular chloride channel in the collecting duct cells of the kidney, a process that requires claudin 8 (CLDN8) for TJ assembly [10, 14, 15]. CLDN8, often termed a barrier-forming claudin, contributes to paracellular barriers that reduce sodium permeability in Madin-Darby canine kidney (MDCK) cells [16]. Various stimuli, such as lipopolysaccharides (LPS), cytokines, pathogens, and ethanol, can alter their expression, localization, and phosphorylation/dephosphorylation. Several signaling pathways involved in the regulation of their barrier function have been reported [10]. Additionally, microRNAs have been found to regulate TJ proteins and modulate epithelial and endothelial barrier function [17]. Given that TJs can be dynamically regulated by multiple signaling pathways, and their effects vary with different triggers, microenvironments, and cell types, identifying the key TJs associated with enhanced pulmonary vascular permeability during viral pneumonia and exploring their regulatory mechanisms can contribute to a deeper understanding of viral pathogenicity.

Here, we hypothesize that TJs of PMVECs might play a significant role in inducing hyperpermeability of the vascular barrier after communication with HP-PRRSV-infected PAMs. Through cytokine screening and functional analyses, we identified that IL-1β and TNF-α, produced by HP-PRRSV-infected PAMs, can communicate with PMVECs. This communication prompts the transcription factor ILF2 to accumulate in the nucleus, along with the expression of *ssc-miR-185*, which inhibits the expression of the tight junction protein CLDN8. Meanwhile, GTF3C2 and THRAP3 enhance the expression of CLDN4 by entering the nucleus and binding to the flank region of the CLDN4 promoter. The dysregulation of both CLDN8 and CLDN4 contributes to the breakdown of the lung vascular endothelial barrier. This study not only explores the molecular details of HP-PRRSV-induced endothelial barrier dysfunction, but may also provide clues for further identifying targets for treating viral pneumonia.

## Materials and methods

### Cells and virus strain

Primary PAMs were collected from alveolar lavage fluid of 6-week-old specific-pathogen-free (SPF) pigs (Beijing Center for SPF Swine Breeding & Management) as previously described [18]. And the PAMs were maintained in RPMI 1640 medium (Gibco, 72400047) supplemented with 10% (v/v) fetal bovine serum (FBS, Gibco, 10099141C), 100 U/ml penicillin, 100 μg/ml streptomycin at 37 °C under a humid 5% CO_2_ atmosphere. PMVECs were purchased from YaJi Biological (YS1234C) and cultured in RPMI 1640 medium supplemented with 5% (v/v) FBS, 100 U/ml penicillin, 100 μg/ml streptomycin at 37 °C under a humid 5% CO_2_ atmosphere. The 8^th^ passage virus of Chinese HP-PRRSV strain JXwn06 (GenBank accession number: EF641008) was used in this study [19]. For virus infection and other related assays, primary PAMs and PMVECs were grown in RPMI 1640 medium supplemented with 2% FBS, 100 U/ml penicillin, and 100 μg/ml streptomycin.

### Animal inoculation experiment

Six healthy 36-day-old landrace pigs were purchased from Beijing Center for SPF Swine Breeding & Management and raised in the animal facilities at China Agricultural University, allowed to acclimatize to the housing facility for 7 days. All of these six pigs were further confirmed to be free of PRRSV, porcine circovirus 2 (PCV2), classical swine fever virus (CSFV), and pseudorabies virus (PRV) by both the corresponding commercial ELISA kits and (RT-) PCR. Then the pigs were randomly divided into two groups (n = 3), which were separately housed in individual rooms. Each pig in the experimental group was intranasally inoculated with 2 ml JXwn06 virus with a titer of 10^5^ TCID_50_/ml, while the pigs in the control group were inoculated with primary PAMs culture supernatant simultaneously. The clinical symptoms and rectal temperature were daily recorded. All the pigs were euthanized and necropsied at the 4 days post-inoculation (dpi), when all inoculated pigs progressed into the early stage of inflammation, manifested as body temperature increased.

### Histopathological examination of lung tissue

The microscopic pathological changes in the lung of each pig were investigated as previously described [20]. Briefly, lung tissue samples were taken and fixed with 4% paraformaldehyde solution at room temperature for 48 h and then processed by conventional histopathological procedures. The section was stained with hematoxylin and eosin (H&E) for microscopic pathological change examination.

### Alveolar lavage fluid (ALF) collection

ALF collection was performed as previously described [21]. Briefly, the lungs collected from each piglet post-mortem were injected with 10 ml of RPMI 1640 medium via their trachea respectively, and then were massaged adequately, yielding about 5 ml of recovery. The supernatants were collected after centrifugation at 2,500 × g at 4 °C for 15 min and followed with sterilization via 0.22 μm filtration. The ALFs were used for treating PMVECs to test trans-endothelial electrical resistance (TEER) and FITC-Dextran assay in vitro immediately or frozen at −80 °C.

### Preparation of conditioned medium (CM)

Primary PAMs were cultured in RPMI 1640 medium supplemented with 10% FBS for 12 h, and then they were inoculated with JXwn06 at a multiplicity of infection (MOI) of 5, or culture medium as mock. After 1 h of incubation, the supernatants were removed and followed with 3 times washes with RPMI 1640 medium, then the cells were further incubated for 24 h in RPMI 1640 medium supplemented with 2% FBS at 37 °C. The medium was then harvested, centrifuged at 2,500 g for 15 min, and filtrated by 0.22 μm filters to eliminate cell debris. The cleared CM was collected and 1:1 diluted before treating the PMVECs.

### In vitro TEER and FITC-Dextran Transwell assay

A total of 1 × 10^5^ PMVECs were seeded onto Transwell apical chambers (12 mm diameter, 0.4 μm pore size, Corning), and grown in RPMI 1640 medium supplemented with 5% FBS to confluence for 2 days. Endothelial cell monolayers were subsequently stimulated for 12 h or 24 h with respective treatments, including ALF, CM, purified JXwn06 viral particles, PRRSV-removed CM, IL-1β (R&D System, 681-PI-010/CF), IL-6 (Abcam, ab209267), TNF-α (Abcam, ab87909), Anakinra (MCE, HY-108841), Tocilizumab (MCE, HY-P9917), or Adalimumab (MCE, HY-P9908). At the indicated time points post-treatment, the medium was replaced by Hank's balanced salt solution (HBSS, Gibco, 14025076). TEER was measured using an Epithelial Volt Ohm Meter (EVOM) with "chopstick" electrodes (Beijing Kingtech Technology) as previously described [6]. For the FITC-Dextran Transwell assay, HBSS mixed with FITC-Dextran (1 mg/ml, average MW 4000, Sigma-Aldrich, 46944) was added to the apical chambers of the Transwell, meanwhile, the medium in the basolateral chamber was replaced by HBSS solution. 1 h later, the paracellular permeability was evaluated by monitoring the endothelial transcellular passage of FITC-Dextran to the basolateral chamber on Spark® fluorescence microplate reader (Tecan Austria GmbH).

### Virus infection and cytokines measurement

The primary PAMs were infected with PRRSV strain JXwn06 at a multiplicity of infection (MOI) of 5. After incubation at 37 °C for 1 h, the unbound viruses were removed by washing three times with serum-free RPMI 1640 medium, and the culture medium was replaced with RPMI 1640 medium containing 2% FBS. At 24 hpi, the culture medium was harvested for virus purification by sucrose density gradient centrifugation or cytokines measurement.

The concentration of pig IL-1β (CSB-E06782p), IL-6 (CSB-E06786p), TNF-α (CSB-E16980p), and TGF-β1 (CSB-E06843p) in the supernatant of PRRSV (MOI = 5) infected-PAMs was quantified using the corresponding ELISA kits supplied by CUSABIO BIOTECH, according to the manufacturer's instructions.

### Blockade of proinflammatory cytokines signaling

To verify the function of IL-1β, IL-6, and TNF-α on the endothelial permeability during the PRRSV infection, the signaling pathways of these three proinflammatory cytokines were blocked by their antagonist. The IL-1 receptor antagonist Anakinra (MCE, HY-108841), and anti-IL-6-receptor mAb Tocilizumab (MCE, HY-P9917), were respectively used to pretreat the PMVECs monolayers for 2 h, with the final concentration of 20 μg/ml in the RPMI 1640 medium containing 2% FBS. Or, the TNF-α neutralizing antibody Adalimumab (MCE, HY-P9908) was added into the CMs from JXwn06- and mock-infected primary PAMs for 2 h. Then, the PMVECs monolayers with or without pretreatment were incubated with the corresponding CM for another 12 h or 24 h, before evaluating their paracellular permeability.

### Transmission electron microscopy (TEM) assay

For TEM, the confluent PMVECs in vitro were fixed with 2.5% glutaraldehyde and 2% paraformaldehyde in 0.1 M sodium cacodylate buffer (pH 7.4) for at least 2 h at room temperature. Then the cells were washed in 0.1 M cacodylate buffer and postfixed with 1% Osmium tetroxide (OsO_4_)/1.5% Potassium ferrocyanide (K4FeCN6) for 1 h, followed by washes in water. After that, the cells were further incubated in 1% aqueous uranyl acetate for 1 h followed by washes in water and subsequent dehydration in grades of alcohol. The samples were then put in propylene oxide for 1 h and infiltrated in a 1:1 mixture of propylene oxide and TAAB Epon (Marivac Canada Inc.). Next, the samples were embedded in TAAB Epon and polymerized at 60 °C for 48 h. The ultrathin sections (about 60 nm) of samples were cut on a Reichert Ultracut-S microtome and picked up to put on the copper grids for staining with lead citrate. Last, they were examined in a JEOL 1400 TEM microscope. Relative intensity of cell junctions PMVECs was quantified by using Image J, in which JXwn06 CM-treated PMVECs was normalized against that in mock CM-treated group.

### Gene overexpression, gene interference, and cell transfection

For gene overexpression experiments, the pig CLDN8, CLDN4, ILF2, GTF3C2, and THRAP3 protein coding region (CDS) were cloned into the pCAGGS-HA expressing vector with the restriction sites *Eco*RI and *Xho*I. A flag tag (DYKDDDDK) was added to the N-terminus of the CLDN8 and CLDN4 CDS, while to the C-terminus of the rest three molecules. PMVECs monolayers were transfected with the recombinant plasmids using Lipofectamine® LTX&PLUS™ reagent (Thermo Fisher, 15338).

For gene knockdown or miRNA relative experiments, silencing RNA (siRNA) duplex (GenePharma Co,. Ltd.), miRNA mimics (GenePharma), or scramble control (NC) duplex was transfected into PMVECs by using Lipofectamine® 2000 reagent (Thermo Fisher, 11668).

### Luciferase reporter assay

The full length of the potential promoter of pig CLDN8 (2,000 bp, Chr13: 193,564,091–93,566,090, namely −2,000 ~ to −1 upstream of the start codon, and the first base of start codon ATG is identified as + 1) and CLDN4 (2,000 bp, Chr3: 11,050,281–11,052,280), as well as their different truncated fragments, were cloned into the pGL3-Enhancer vector (Promega, E1771), upstreaming the luciferase gene at the *Xho*I and *Hin*dIII restriction sites. For cell transfection, 1 × 10^5^ cells/well were seeded into 24-well plates. After 12 h, the reporter plasmid or empty vector was co-transfected with the pRL-TK reporter vector into PMVECs, by using Lipofectamine® LTX&PLUS™ reagent. The transfected cells were incubated for 36 h and then treated with JXwn06 CM for another 6 h and the total protein from PMVECs was extracted by Passive Lysis Buffer, last, the luciferase activity was examined by using Dual-Luciferase® Reporter Assay System (Promega, E1910) on GloMax. Firefly luciferase activity was normalized against Renilla luciferase values, and the ratio of Firefly/Renilla luciferase activity was presented.

To confirm the direct regulation of *ssc-miR-185* on CLDN8, the wild type protein-coding region (CDS) of CLDN8 (CLDN8-CDS-Wt) or the mutated seed sequence of *ssc-miR-185* in CLDN8 CDS (CLDN8-CDS-Mut) was cloned into the pGL3-Control vector (Promega, E1741) at the downstream of the luciferase gene with the restriction site *Xba*I. About 1 × 10^5^ cells/well were first seeded into 24-well plates, and 12 h later, the recombinant plasmid CLDN8-CDS-Wt or CLDN8-CDS-Mut was co-transfected into PMVECs together with pRL-TK reporter vector, as well as *ssc-miR-185* mimic, or its negative control (NC) by using Lipofectamine® 2000 reagent. After 36 h, the relative luciferase activity was measured with the Dual-Luciferase® Reporter Assay System.

### DNA pulldown assay

DNA pull-down assay was carried out as previously described [22]. Briefly, PMVECs cultured in a 100 mm petri dish were treated with CMs from JXwn06- and mock-infected primary PAMs for 12 h, and then washed twice with ice-cold PBS. The cells were scraped off from the plates and harvested by centrifugation at 1,000 × g for 5 min at 4 °C. The nuclear protein was extracted by using the Nuclear and Cytoplasmic Protein Extraction Kit (Beyotime, P0027) by following the manufacturer's protocol. 1 mg nuclear protein was transferred to a fresh microcentrifuge tube and sonicated salmon sperm DNA (Sigma-Aldrich, D1626) was added with the final concentration of 100 μg/ml to block unspecific DNA–protein interactions. The samples were incubated on a rotator for 15 min at room temperature. Double-stranded DNA probes were amplified from pGL3-Enhancer-CLDN8 −1,000 to −1 plasmid, pGL3-Enhancer-CLDN4 −2,000 to  −1 plasmid, and pGL3-Enhancer-CLDN4 −500 ~ to −1 plasmid by the 5'-biotin labeled reverse primer, respectively. DNA gel electrophoresis was employed to completely separate the probe from any primer dimers, and the band was cut out and purified by E.Z.N.A.® Gel Extraction Kit (OMEGA, D2500) following the manufacturer’s instruction. 1 μg of DNA probe was added into 1 mg of nuclear extract, and then incubation on a rotator for 30 min at room temperature. Afterward, streptavidin-coated magnetic beads were added to the mixture and incubated for 5 min at room temperature. The combined proteins were electrophoresed on SDS-PAGE followed by Coomassie blue staining and mass spectrometry analysis. The interaction network of candidates that bind to CLDN8’s or CLDN4’s promoters were conducted by STRING (Version: 12.0, https://cn.string-db.org/).

### Immunofluorescence assay

PMVECs grown to ~ 30% confluence on coverslips in 12-well plates were stimulated with CMs prepared from JXwn06- or mock-infected primary PAMs for ~ 12 h, respectively. Then the transfected cells were fixed in 4% paraformaldehyde (w/v) in PBS for 15 min at room temperature. All subsequent steps were performed at room temperature. Cells were permeabilized with 0.1% (v/v) Triton X-100 for 10 min and blocked with 2% (w/v) bovine serum albumin (BSA) in PBS for 30 min. Then target proteins were immuno-stained by primary Mouse anti-ILF2 antibody (2 μg/ml, Proteintech, 14714-1-AP) or Rabbit anti-THRAP3 (2 μg/ml, Sigma-Aldrich, HPA063765) and subsequently Alexa Fluor 488 (568)-conjugated Goat anti-mouse (rabbit) IgG (H + L) F(ab′)2 fragment antibody (Thermo Fisher, 11017/11011), which were both diluted in PBS with 2% BSA (w/v). And the incubation time was 1 h for both steps. Cell nuclei were stained with DAPI (Beyotime, C1005) for 3 min. The coverslips were mounted on microscope slides with Aqua-Poly/Mount (Polysciences, 18606-20), and the fluorescent images were captured with the Nikon A1 confocal microscope.

### RT-qPCR

For mRNA expression detection, total RNA was extracted from cultured cells using Trizol (Thermo Fisher, 15596026) and the quality and integrity of the RNA were tested. Then the RNA was reversed-transcribed by using the FastKing RT Kit (With gDNase) (TIANGEN, KR116). The 1:3 diluted cDNA was used as templates for the qPCR, which was performed in Applied Biosystems SYBR™ Select Master Mix (Thermo Fisher, 4472908) or CFX96 Real-Time PCR detection system (Bio-Rad). The expression of each target mRNA relative to β-actin was calculated based on the cycle threshold (Ct) as 2^−Δ (ΔCt)^, in which ΔCt = Ct (target)−Ct (β-actin), and Δ (ΔCt) = ΔCt (test sample)−ΔCt (control sample).

For detecting miRNA expression, RT-qPCR was performed as the manual of miRcute Plus miRNA First-Strand cDNA Kit (TIANGEN, KR211) and miRcute Plus miRNA qPCR Kit (SYBR Green) (TIANGEN, FP411), with the primers listed in Table 1.

**Table 1:** List of primers used in this study

Primer Name	Primer Sequence (5′-3′)	Length of amplicon (bp)
For real-time PCR		
CLDN8-F^a^	TGGTGGTGTTGGAATGGTGG	
CLDN8-R^b^	GTTGCTTCCAATGAAGGCGG	
CLDN4-F	TGGATGATGAGAGCGCCAAG	
CLDN4-R	GGGATTGTAGAAGTCGCGGA	
ILF2-F	GTCAAGCCAGCACCTGATGA	
ILF2-R	TGGAGCCACGATCAAGTTGT	
GTF3C2-F	AGAGCTCTCAACAACCCTGC	
GTF3C2-R	TCATCCTGTGCCGTGTCATC	
THRAP3-F	TGACACGGCACAGGATGAAG	
THRAP3-R	GGTAAGCCGTTAGGAGCCAC	
β-actin-F	ACCACCATGTACCCAGGCAT	
β-actin-R	GGACTCGTCGTACTCCTGCT	
*ssc-miR-185*-F	CGCGTGGAGAGAAAGGCAGT	
*ssc-miR-432-3p*-F	GCGTGGATGGCTCCTCC	
*ssc-miR-9813-5p*-F	CGCGGAAGGTCGGAGG	
U6-F	CTCGCTTCGGCAGCACATA	
For genes clone		
Flag-CLDN8-F	^*c*^GATTACAAGGATGACGATGACAAGATGGCCAGCAATGCCCTG	702
Flag-CLDN8-R	CTATACATACTGACTTCTG	
Flag-CLDN4-F	GATTACAAGGATGACGATGACAAGATGGCTTCCATGGGGCTG	654
Flag-CLDN4-R	TTACACGTAGTTGCTGGCGGG	
*Eco*RI-Flag-CLDN8-F	^*d*^gttccagattacgctgaattcGATTACAAGGATGACGATGACAAGA	744
Flag-CLDN8-*Xho*I-R	attaagatctgctagctcgagCTATACATACTGACTTCTGGAGTACACACTC	
*Eco*RII-Flag-CLDN4-F	gttccagattacgctgaattcGATTACAAGGATGACGATGACAAGA	696
Flag-CLDN4-*Xho*I-R	attaagatctgctagctcgagTTACACGTAGTTGCTGGCGGG	
ILF2-Flag-F	ATGAGGGGGGACAGAGGCC	1197
ILF2-Flag-R	TCACTTATCGTCGTCATCCTTGTAATCCTCCTGAGTCTCCATGCTTTC	
GTF3C2-Flag-F	ATGGATACCTACGGGGTCGG	2739
GTF3C2-Flag-R	CTACTTATCGTCGTCATCCTTGTAATCGGGACTGGGCAGAAGGCGATG	
THRAP3-Flag-F	ATGTCAAAAACAAACAAATCTAAGTCTG	2901
THRAP3-Flag-R	CTACTTATCGTCGTCATCCTTGTAATCCTCAGTTGTGGGCTGCAAATT	
*Eco*RI-ILF2-Flag-F	gttccagattacgctgaattcATGAGGGGGGACAGAGGCC	1239
ILF2-Flag-*Xho*I-R	attaagatctgctagctcgagTCACTTGTCATCGTCATCCTTGTAA	
*Eco*RI-GTF3C2-F	gttccagattacgctgaattcATGGATACCTACGGGGTCGG	2781
GTF3C2-Flag-*Xho*I-R	attaagatctgctagctcgagCTACTTGTCATCGTCATCCTTGTAATC	
*Eco*RI-THRAP3-Flag-F	gttccagattacgctgaattcATGTCAAAAACAAACAAATCTAAGTCTG	2943
THRAP3-Flag-*Xho*I-R	attaagatctgctagctcgagCTACTTGTCATCGTCATCCTTGTAATC	
pGL3-Control-CLDN8 CDS reporter plasmid construction		
*Xba*I-CLDN8-F	agatcgccgtgtaattctagaATGGCCAGCAATGCCCTG	720
CLDN8-R-*Xba*I	gccggccgccccgactctagaCTATACATACTGACTTCTGGAGTACACACTC	
*Xba*I-CLDN8-Mut1-F	agatcgccgtgtaattctagaATGGCCAGCAATGCCCTG	252
CLDN8-Mut1-R	^*e*^taggtccttctcttg**TC**C**GTC**CAGGGAATCGTAGA	
CLDN8-Mut2-F	acggaC**AAGAGAA**GGACCTACAGGCGT	488
CLDN8-Mut2-R-*Xba*I	gccggccgccccgactctagaCTATACATACTGACTTCTGGAGTACACACTC	

^a^F Represents forward PCR primer

^b^R represents reverse PCR primer

^c^The sequences of the Flag gene are underlined

^d^Homologous sequences are lowercased

^e^The mutation sites are marked in **bold**

### Western blot

The cells were harvested and lysed on ice by RIPA Lysis Buffer (Beyotime, P0013B) containing protease inhibitor (Beyotime, ST506). And the lysates were centrifuged at 10,000 × g at 4 °C for 15 min to remove the cell debris. The concentration of the whole cellular lysis was quantified by Pierce™ BCA Protein Assay Kit (Thermo Fisher, 23225) following the manufacturer’s instructions. Cell lysates were heat-denatured and separated by SDS-PAGE, blotted onto PVDF membranes, which were blocked with 5% skim milk rotating for 2 h at room temperature. The membrane was probed against the target proteins by incubation with primary antibody of Rabbit anti-CLDN8 (1.0 μg/ml, Novus Biologicals, NBP1-59157), Rabbit anti-CLDN4 (1:1,000, Abcam, ab53156), Mouse anti-Flag (0.1 μg/ml, Medical & Biological Laboratories, M185), or Mouse anti-β-actin (1:5,000, CUSABIO BIOTECH, CSB-MA000187), rotating overnight at 4 °C, subsequently secondary Goat anti-Rabbit antibody (1:10,000, Beyotime, A0208) or Goat anti-Mouse antibody (1:10,000, Beyotime, A0216) coupled to horseradish peroxidase rotating for 1 h at room temperature. Then the bands were visualized using the Pierce ECL Western blot substrate (Thermo Fisher, 32209) and the images were taken by using the Bio-Rad ChemiDoc Touch system (Bio-Rad).

### Statistical analysis

GraphPad Prism 10.1.2 software was used to perform statistical analysis. Two-tailed Student’s test was used to test the differences between two groups, and the differences among multiple groups were analyzed by two-tailed unpaired Student’s t-test or one-way ANOVA. Data are shown as mean ± SD of biological triplicates. Details of the statistical analysis for each experiment as indicated in the relevant figure legends. For all statistical tests, *p* values < 0.05 were considered statistically significant. Except for DNA pulldown, all experiments above have triple biological repeated at least.

## Results

### HP-PRRSV infection enhances pulmonary vascular permeability

**Fig. 1:**
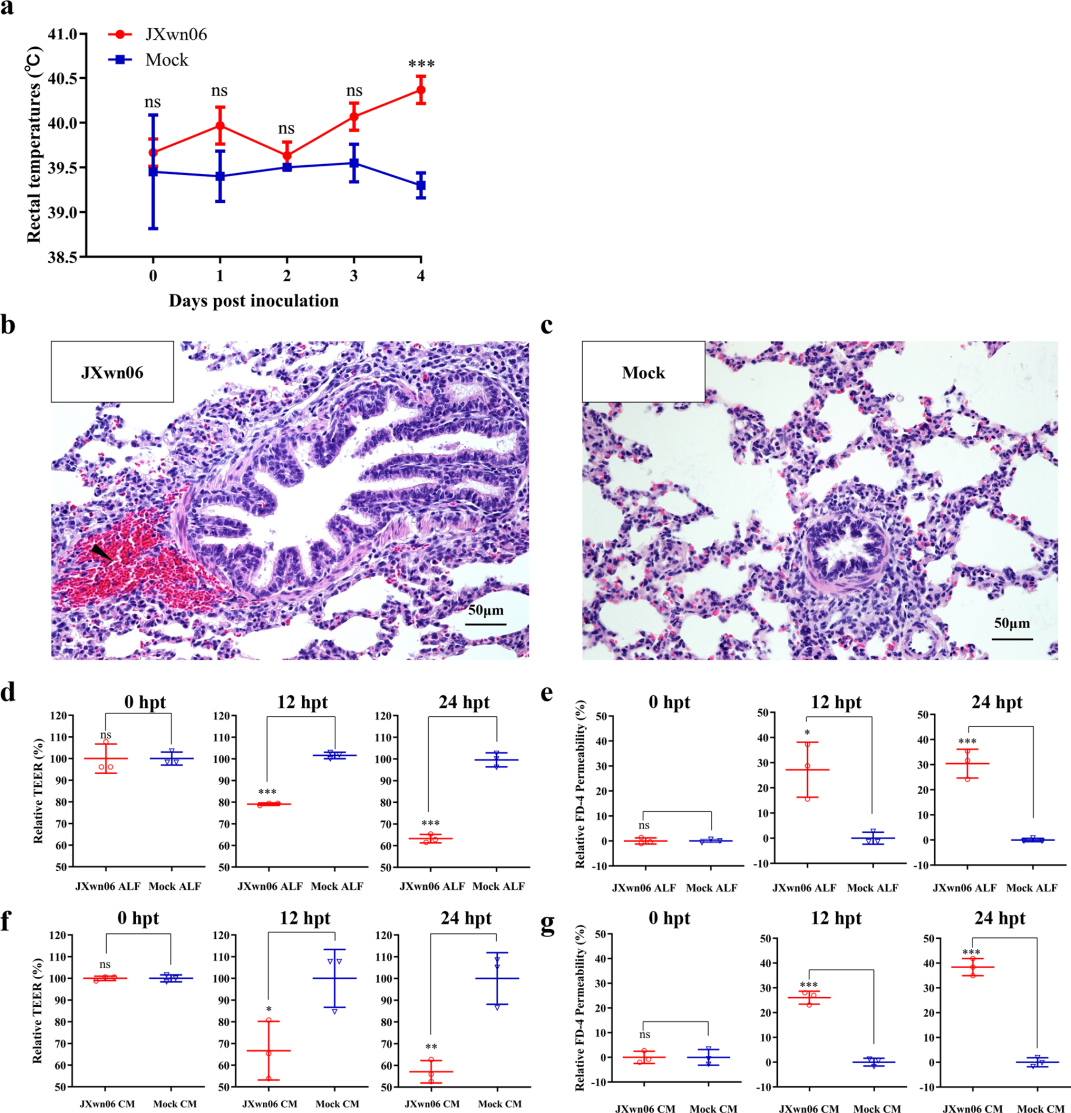
HP-PRRSV strain JXwn06 infection induces pulmonary microvascular hyperpermeability in vivo and in vitro. **a** The rectal temperature of pigs in the JXwn06-inoculated group or Mock. JXwn06 induced significantly higher body temperature at 4 dpi (*p* < 0.001). **b**, **c** The representative microscopic lung lesions of pigs euthanized at 4 dpi. The section was stained with hematoxylin and eosin (H&E). A solid triangle in the left panel manifests hemorrhage around the bronchiole. **d** JXwn06 ALF induced the TEER reduction of pulmonary endothelial cells at both 12 hpt (*p* < 0.001; middle panel) and 24 hpt (*p* < 0.001; right panel). **e** FITC-labeled Dextran permeability of PMVECs was significantly increased by treating with JXwn06 ALF at both 12 hpt (*p* < 0.05; middle panel) and 24 hpt (*p* < 0.001; right panel). **f** JXwn06 CM induced the TEER reduction of pulmonary endothelial cells at both 12 hpt (*p* < 0.05; middle panel) and 24 hpt (*p* < 0.01; right panel). **g** FITC-labeled Dextran permeability of PMVECs was significantly increased by treating with JXwn06 CM at both 12 hpt (*p* < 0.001; middle panel) and 24 hpt (*p* < 0.001; right panel).The data are shown as means ± SD (error bars), n = 3 independent experiments were performed in triplicate. Asterisks indicate statistical significance (ns, *p* > 0.05; *, *p* < 0.05; **, *p* < 0.01; ***, *p* < 0.001)

To investigate the effects of HP-PRRSV on pulmonary vascular permeability, microscopic pathological lung lesions were examined at 4 days post-inoculation (dpi), a time when the body temperature of all pigs in the JXwn06 (HP-PRRSV) inoculated group had increased (*p* < 0.001; Fig. 1a). Extensive hemorrhage, as well as infiltration of inflammatory cells and serous fluid in the lung vascular system, indicated enhanced pulmonary vascular permeability in HP-PRRSV inoculated pigs (Fig. 1b and c). To further confirm that HP-PRRSV infection enhances pulmonary vascular permeability, an in vitro model using the Transwell cell culture system was employed. Pig PMVECs monolayers were grown on the apical chambers of Transwell culture inserts, and alveolar lavage fluid (ALF) or conditioned medium (CM) derived from JXwn06-infected primary PAMs was added to the chambers. Subsequently, trans-endothelial electrical resistance (TEER) and FITC-Dextran permeability were assessed after treatment. As shown in Fig. 1d and e, ALF collected from JXwn06-infected pigs (referred to as JXwn06 ALF) caused a nearly 20% to 40% reduction in TEER (*p* < 0.001 and *p* < 0.001 respectively; Fig. 1d) and an up to 30% increase in FITC-Dextran permeability (*p* > 0.05 and *p* < 0.001 respectively; Fig. 1e), compared to the mock infection group, after 12 or 24 h of ALF treatment. Concurrently, the trends in TEER (Fig. 1f) and FITC-Dextran permeability (Fig. 1g) of PMVECs cultured with CM derived from JXwn06-infected primary PAMs (referred to as JXwn06 CM) were similar to those treated with JXwn06 ALF.

### PRRSV particles do not directly enhance the permeability

**Fig. 2:**
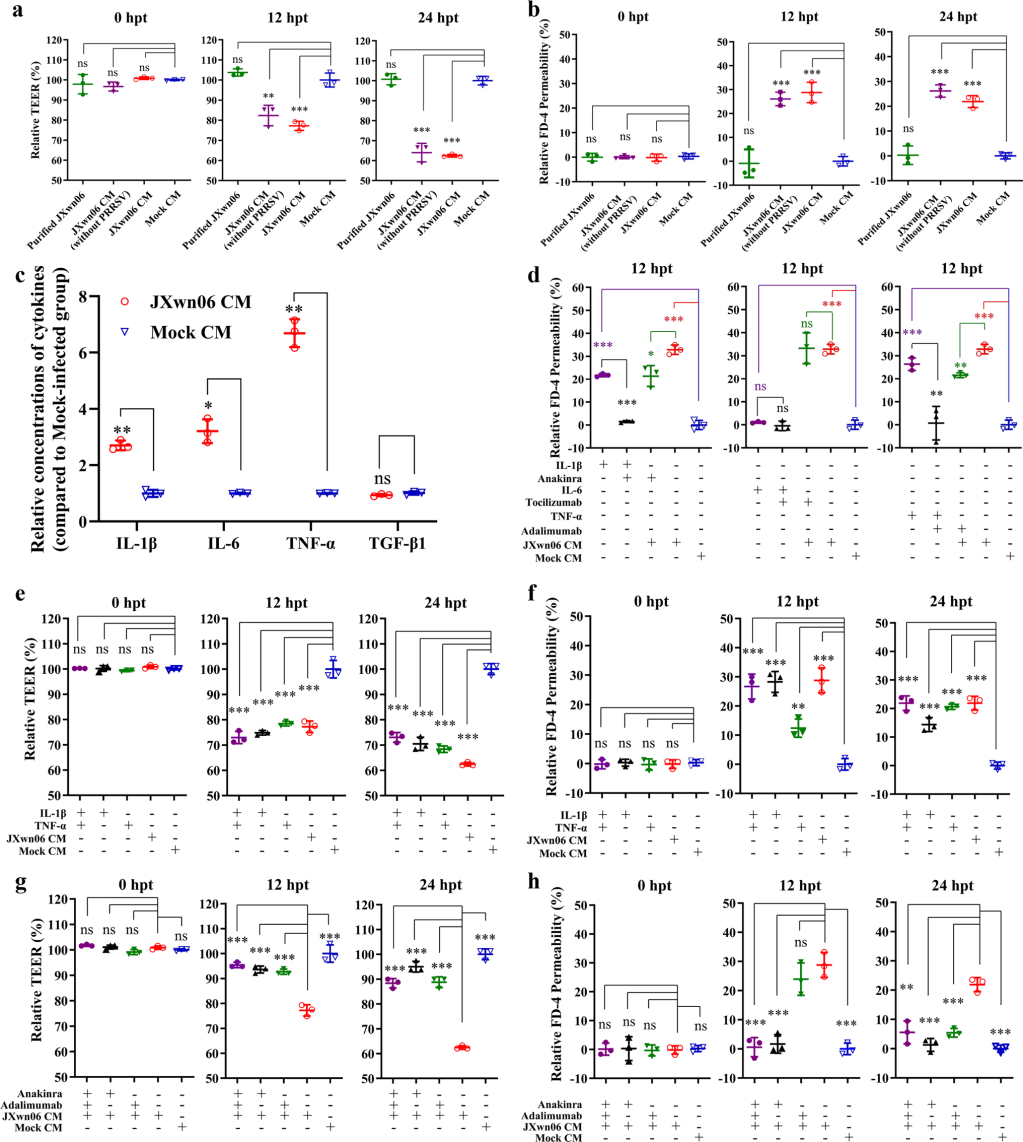
IL-1β and TNF-α released from JXwn06-infected PAMs are responsible for the hyperpermeability of the PMVECs barrier. **a** PRRSV particles purified by sucrose density gradient centrifugation (represented by green solid circle) minimally affected PMVECs’ TEER at any time point (*p* > 0.05), while virus-removed JXwn06 CM (represented by purple solid triangle) induced similar changes in the TEER as JXwn06 CM did at both 12 hpt (*p* < 0.01; middle panel) and 24 hpt (*p* < 0.001; middle panel). **b** The same as (**a**), excepted that FITC-labeled Dextran permeability was analyzed. Purified PRRSV particles have limited effects on PMVECs’ permeability at any time point (*p* > 0.05), while virus-removed JXwn06 CM increased PMVECs’ permeability to the same extent of JXwn06 CM at the indicated time points (both *p* < 0.001). **c** The concentrations of IL-1β, IL-6, TNF-α, and TGF-β1 in the JXwn06 CM were detected by the corresponding ELISA kits and shown as relative folds compared with the concentration of mock CM (*p* < 0.01, *p* < 0.05, *p* < 0.01 and *p* > 0.05 respectively). **d** The bioactivity of recombinant porcine IL-1β, IL-6, TNF-α and their corresponding antagonist reagents Anakinra, Tocilizuman, and Adalimuab (10 μg/ml) was validated by measuring the FITC-Dextran permeability of PMVECs. **e**, **f** Detecting the effects of IL-1β and TNF-α on pulmonary endothelial hyperpermeability. Recombinant porcine IL-1β and TNF-α proteins were added individually or together into PMVECs’ culture medium, and then TEER (**e**) and FITC-Dextran permeability (**f**) were measured. The JXwn06 CM and mock CM were set as the positive and negative control. On the contrary, these effects caused by JXwn06 CM can be abolished by pre-treating PMVECs with Anakinra and neutralizing TNF-α by Adalimumab (20 μg/ml), the corresponding antagonist reagents of IL-1β and TNF-α (**g** and **h**). The data are shown as means ± SD (error bars), n = 3 independent experiments were performed in triplicate. Asterisks indicate statistical significance (ns, *p* > 0.05; *, *p* < 0.05; **, *p* < 0.01; ***, *p* < 0.001)

To determine whether PRRSV virus particles or factors released from PAMs are responsible for pulmonary vascular hyperpermeability, purified virus particles of JXwn06 obtained through sucrose density gradient centrifugation, and virus-removed supernatant (CM) from JXwn06-infected PAMs, where the virus was removed by high-speed centrifugation, were used to stimulate PMVECs separately. Compared to the mock CM, which served as a negative control, PRRSV particles had a minimal effect on TEER (*p* > 0.05; Fig. 2a) and FITC-Dextran permeability (*p* > 0.05; Fig. 2b). In contrast, the virus-removed JXwn06 CM affected TEER (*p* < 0.001; Fig. 2a) and FITC-Dextran permeability to the same extent as the JXwn06 CM (*p* < 0.001; Fig. 2b). Thus, the hyperpermeability of PMVECs was attributed to soluble mediators released by HP-PRRSV-infected PAMs, rather than direct stimulation by virus particles.

### HP-PRRSV enhances pulmonary vascular permeability through IL-1β and TNF-α released from virus-infected PAMs

To further identify the molecules responsible for pulmonary vascular hyperpermeability, the concentrations of three proinflammatory factors (IL-1β, IL-6, TNF-α) and TGF-β1 in JXwn06 or mock conditioned medium (CM) were determined using ELISA kits. Except for TGF-β1 (*p* > 0.05), the other three cytokines were significantly upregulated in JXwn06 CM compared to mock CM (*p* < 0.01, *p* < 0.05, *p* < 0.01, respectively) (Fig. 2c). The effects of these cytokines on PMVECs monolayer permeability were evaluated by adding recombinant porcine IL-1β, IL-6, or TNF-α proteins to the cell culture medium or by using their antagonists to block the cytokines from JXwn06 CM. The results indicated that recombinant pig IL-1β and TNF-α (*p* < 0.001, *p* < 0.001, respectively; Fig. 2d) proteins individually induced a 20% to 28% increase in FITC-Dextran permeability, similar to the effect of JXwn06 CM, which induced more than a 30% increase. The antagonist reagents Anakinra (for IL-1β) and Adalimumab (for TNF-α) specifically blocked the function of IL-1β and TNF-α (*p* < 0.05, *p* < 0.01, respectively; Fig. 2d), partially mitigating the permeability changes induced by JXwn06 CM. In contrast, IL-6 did not affect FITC-Dextran permeability, and its receptor mAb Tocilizumab did not block the permeability effect of JXwn06 CM (*p* > 0.05, *p* > 0.05, respectively; Fig. 2d). Subsequently, the individual and combined effects of IL-1β and TNF-α, along with their antagonist reagents, on TEER and FITC-Dextran permeability were investigated at 0 hpt, 12 hpt, and 24 hpt. The combined action of IL-1β and TNF-α mimicked the effect of JXwn06 CM on PMVECs permeability, reducing TEER and increasing FITC-Dextran permeability (*p* < 0.001, *p* < 0.001, respectively; Fig. 2e and f). The antagonist reagents significantly blocked this effect, with the co-treatment of Anakinra and Adalimumab almost completely reversing the permeability changes at 12 hpt (*p* < 0.001, *p* < 0.001, respectively; Fig. 2g and h).

### Tight junction proteins CLDN8 and CLDN4 are dysregulated in PMVECs

**Fig. 3:**
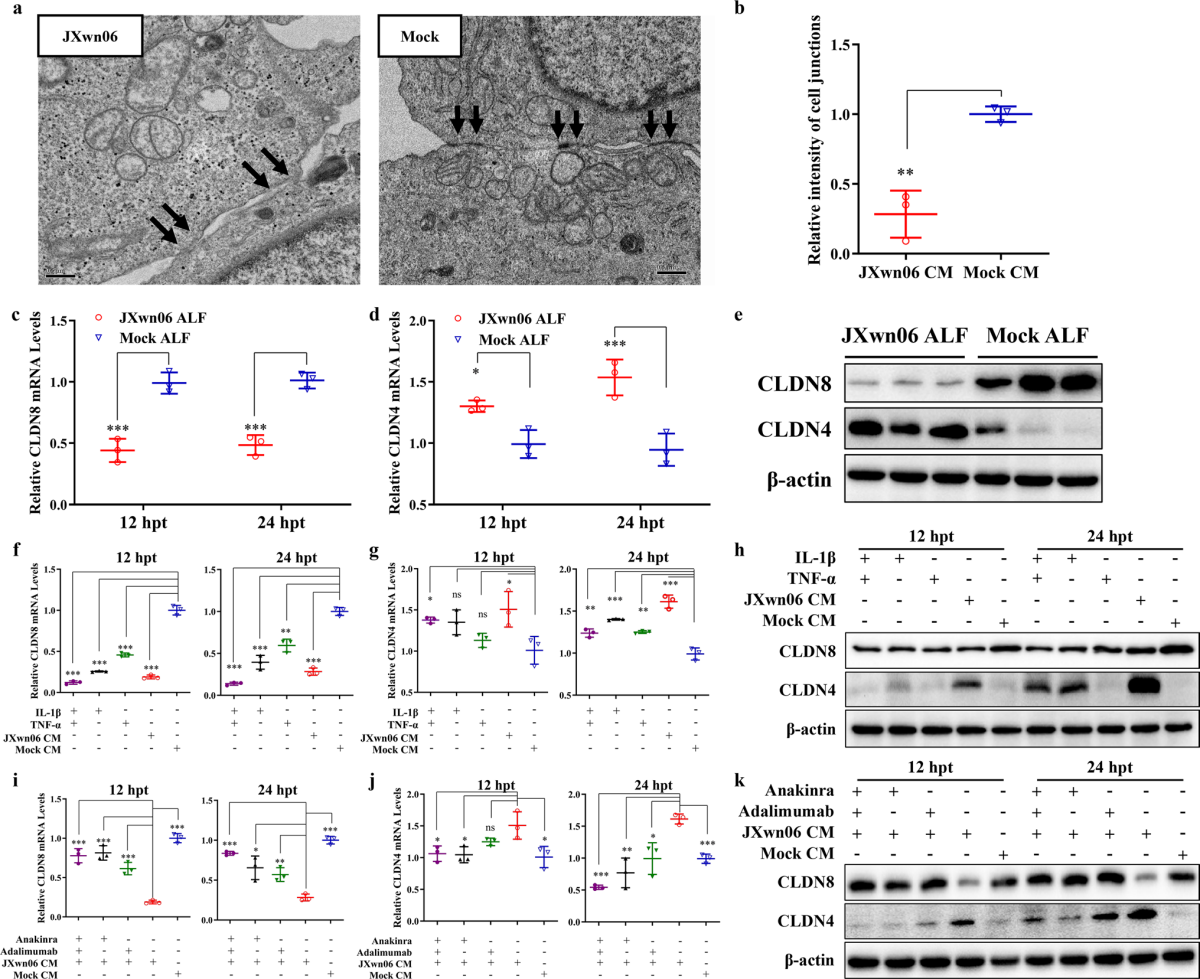
Tight junction proteins CLDN8 and CLDN4 are dysregulated by IL-1β and TNF-α. **a**, **b** Endothelial intercellular junctional integrity in the JXwn06 CM- versus mock CM-treated PMVECs was determined by TEM (bar, 0.5 μm). The relative intensity of cell junctions was quantified by using Image J to analyze 3 TEM pictures in each group (n = 3). Arrows show the region of cell–cell junctions. **c**, **d** Relative abundance of CLDN8 (**c**) and CLDN4 (**d**) mRNA in PMVECs treated with JXwn06 ALF at different time points. The level of target gene mRNA was normalized against β-actin and then compared to the mock ALF-treated group. **e** Western Blotting analysis of CLDN8 and CLDN4 protein levels in PMVECs treated with JXwn06 ALF. β-actin served as the loading control. **f**–**k** Analysis of the effects of IL-1β and TNF-α on the dysregulation of CLDN8 and CLDN4. Recombinant porcine IL-1β and TNF-α proteins were added individually or together to PMVECs’ culture medium, and then the relative mRNA levels of CLDN8 (**f**) and CLDN4 (**g**), as well as their protein levels (**h**) were measured. On the contrary, these effects caused by JXwn06 CM were abolished by pre-treating PMVECs with Anakinra and neutralizing TNF-α with Adalimumab (**i**–**k**). The data are shown as means ± SD (error bars), n = 3 independent experiments were performed in triplicate. Asterisks indicate statistical significance (ns, *p* > 0.05; *, *p* < 0.05; **, *p* < 0.01; ***, *p* < 0.001)

Our latest transcriptomics study has suggested that the mRNA transcription of tight junction (TJ) proteins CLDN8 and CLDN4 in PMVECs is dysregulated upon communication with HP-PRRSV-infected PAMs through paracrine signaling [6]. To further confirm whether the intercellular junctions of PMVECs were disturbed during treatment with JXwn06 CM, confluent PMVECs were subjected to transmission electron microscopy (TEM) analysis. Junctional complexes were easily detectable at the intercellular junctions of neighboring cells treated with mock CM, but in the JXwn06 CM-treated group, many cell–cell junctions were lost, and increased gaps between neighboring membranes were observed (*p* < 0.01; Fig. 3a and b). Then the repetitive experiment carried out by using JXwn06 ALF confirmed the previous conclusion that CLDN8 was downregulated while CLDN4 was upregulated in this test (*p* < 0.001, *p* < 0.001, respectively; Fig. 3c–e). Additionally, to determine if the IL-1β and TNF-α in JXwn06 CM are responsible for the regulation of CLDN8 and CLDN4, the mRNA and protein levels of these two TJs in PMVECs were analyzed using qPCR and Western blot after treatment with these cytokines. Similar to the permeability tests above, the individual and combined effects of IL-1β and TNF-α on the regulation of CLDN8 and CLDN4 were compared with those of JXwn06 CM and mock CM, and the blocking effects of their antagonist reagents on JXwn06 CM-induced changes were also assessed. As shown in Fig. 3f–h, either IL-1β or TNF-α could individually downregulate CLDN8 (significantly at 12 hpt and 24 hpt; *p* < 0.001, *p* < 0.001, respectively) and upregulate CLDN4 (significantly at 24 hpt, *p* < 0.001), with their combined effect closely resembling that of JXwn06 CM. Meanwhile, inhibiting the signaling pathways of IL-1β and TNF-α could prevent the downregulation of CLDN8 and upregulation of CLDN4 induced by treatment with JXwn06 CM (*p* < 0.001, *p* < 0.001, respectively; Fig. 3i–k).

### CLDN8 and CLDN4 are involved in regulating pulmonary vascular permeability

**Fig. 4:**
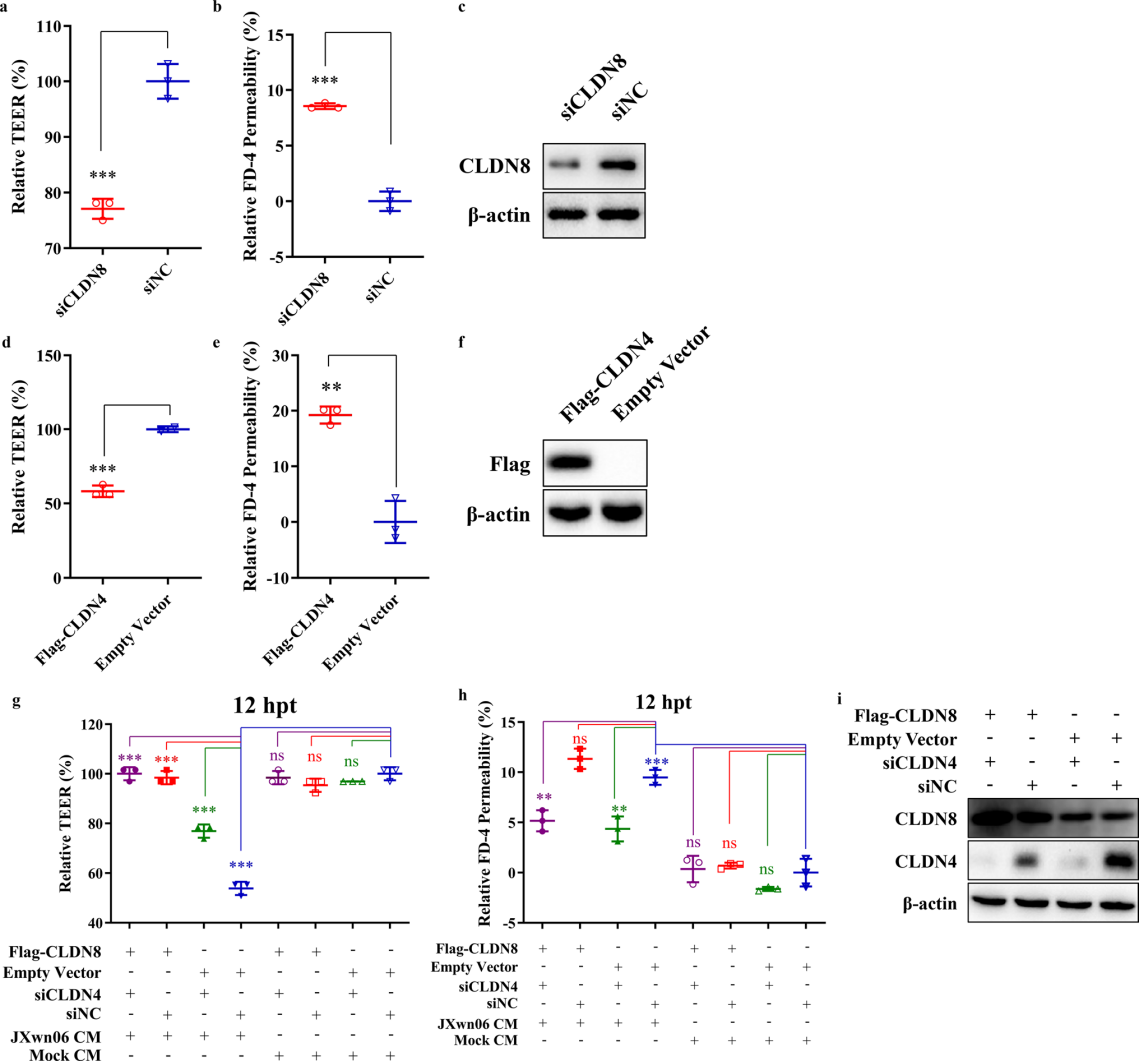
Overexpression of CLDN8 and knockdown of CLDN4 can restore the permeability induced by JXwn06 CM. **a**–**c** PMVECs were transfected with siRNA targeting CLDN8 (siCLDN8) or scrambled siRNA (siNC). At the 36 h post-transfection, the TEER (**a**) and FITC-Dextran permeability (**b**) were measured, and knockdown efficiency of CLDN8 was analyzed by Western blotting (**c**). **d**–**f** PMVECs were transfected with Flag-tagged CLDN4 (Flag-CLDN4) or empty vector. At the 36 h post-transfection, the TEER (**d**) and FITC-Dextran permeability (**e**) were measured, and the overexpression efficiency of CLDN4 was analyzed by Western blotting (**f**). **g**–**i** PMVECs were transfected with Flag-tagged CLDN8 or empty vector, together with siRNA targeting CLDN4 (siCLDN4) or scrambled siRNA (siNC). At the 24 h post-transfection, the cells were further treated with JXwn06 CM, and then the TEER (**g**) and FITC-Dextran permeability (**h**) were measured at the 12 h post-treatment. The data are shown as means ± SD (error bars), n = 3 independent experiments were performed in triplicate. **i** Confirm the overexpression of Flag-tagged-CLDN8 and knockdown of CLDN4 by Western Blotting. β-actin served as the loading control. Asterisks indicate statistical significance (ns, *p* > 0.05; **, *p* < 0.01; ***, *p* < 0.001)

Given that tight junctions (TJs) are critical factors for maintaining the permeability of the vascular endothelium [10], the role of CLDN8 and CLDN4 dysregulation in HP-PRRSV-induced hyperpermeability of the vascular endothelium was further investigated. Initially, as in the permeability testing experiments described above, trans-endothelial electrical resistance (TEER) and FITC-Dextran permeability were used as parameters to assess the impact of downregulated CLDN8 and upregulated CLDN4 on endothelial permeability. Both knockdown of CLDN8 (Fig. 4a–c) and overexpression of CLDN4 (Fig. 4d–f) significantly reduced TEER (*p* < 0.001, *p* < 0.001, respectively) and increased FITC-Dextran permeability (*p* < 0.001, *p* < 0.01, respectively), although the effect of CLDN8 on permeability was relatively minor (less than 10%).

Subsequently, the antagonistic effects of CLDN8 overexpression and CLDN4 knockdown on the permeability changes induced by JXwn06 CM were evaluated (Fig. 4g–i). As shown in Fig. 4g, both CLDN8 overexpression and CLDN4 knockdown could prevent the reduction of TEER caused by JXwn06 CM (*p* < 0.001, *p* < 0.001, respectively). CLDN4 knockdown could also mitigate the increase in FITC-Dextran permeability induced by JXwn06 CM (*p* < 0.01; Fig. 4h), while the effect of CLDN8 overexpression was limited in reducing permeability (*p* > 0.05; Fig. 4h). However, the combined overexpression of CLDN8 and knockdown of CLDN4 could completely counteract the effects of JXwn06 CM (*p* < 0.001, *p* < 0.01, respectively; Fig. 4g and h). In contrast, for PMVECs treated with mock CM, neither CLDN8 overexpression nor CLDN4 knockdown had a significant effect on TEER or FITC-Dextran permeability (Fig. 4g–h). These findings suggest that the dysregulation of TJ proteins CLDN8 and CLDN4 is strongly associated with the pulmonary vascular hyperpermeability caused by HP-PRRSV.

### Regulation factor ILF2 inhibits the transcription of CLDN8 by binding its promoter

**Fig. 5:**
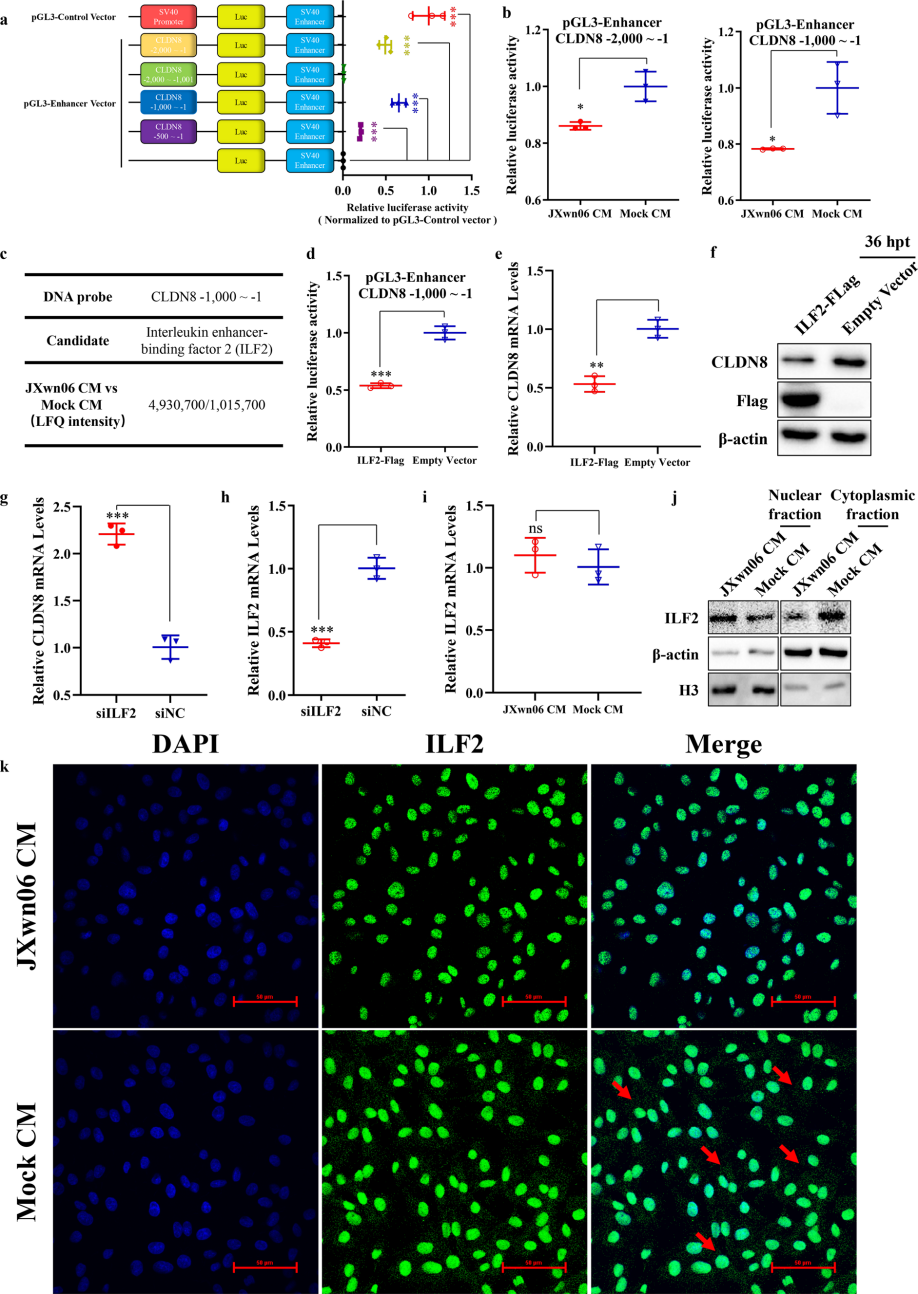
Identify ILF2 that inhibits the transcription level of CLDN8 by entering the nucleus and binding to CLDN8’s promoter. **a** The schematic diagram for construction of the potential promoter regions of CLDN8, and detecting their abilities to initiate the transcription of the reporter gene. **b** The activity of two effective promotor regions of CLDN8 was further evaluated in PMVECs treated with JXwn06 CM or mock CM. **c** ILF2 was enriched in JXwn06 CM treated group, compared with the mock CM treated group, as detected by DNA pulldown and MS analysis. **d** Relative luciferase activity in Flag-tagged ILF2 (ILF2-Flag) transfected PMVECs. **e** Relative abundance of CLDN8 mRNA in ILF2-Flag transfected PMVECs. The level of CLDN8 mRNA was normalized against β-actin and then compared to the empty vector group. **f** Western blotting analysis of CLDN8 protein level in PMVECs transfected with ILF2-Flag. β-actin served as the loading control. **g**, **h** Relative abundance of CLDN8 mRNA (**g**) in the PMVECs with ILF2 knockdown. The knockdown efficiency of ILF2 was confirmed by qPCR (**h**). **i** Relative abundance of ILF2 mRNA in JXwn06 CM treated PMVECs. **j** PMVECs were stimulated with JXwn06 or mock CM, at the 6 h post-treatment, and the cell cytoplasmic and nuclear fractions were prepared and assayed for the presence of ILF2. β-actin and H3 were used as fractionation quality control. **k** The location of endogenous ILF2 in JXwn06 CM treated PMVECs. Mock CM treated PMVECs served as the negative control. JXwn06 CM facilitates endogenous ILF2 accumulated in the nucleus (red arrows show endogenous ILF2 located in the cytoplasm). The fluorescent images were captured with the Nikon A1 confocal microscope (Scale bar, 50 μm). The data are shown as means ± SD (error bars), n = 3 independent experiments were performed in triplicate. Statistical analyses were performed by two-tailed Student’s t-test, and asterisks indicate statistical significance (ns, *p* > 0.05; *, *p* < 0.05; **, *p* < 0.01; ***, *p* < 0.001)

The results suggest that the dysregulation of CLDN8 and CLDN4, caused by IL-1β and TNF-α released from HP-PRRSV-infected PAMs, is responsible for the hyperpermeability of the pulmonary vascular endothelium. However, the underlying mechanism of transcriptional regulation is not well understood. We hypothesized that certain factors might bind to the CLDN8 promoter in the cellular nucleus to downregulate its transcription. To identify these factors, the transcriptional promoter of CLDN8 was first identified by cloning the 5′ flanking region (from −,2000 to −1 bp, putative full-length promoter) from PMVECs into the upstream of the luciferase CDS (*luc* +) in the pGL3-Enhancer vector. This vector contains an SV40 enhancer downstream of *luc* + but lacks eukaryotic promoter sequences. Additionally, three plasmids with truncated versions of the putative promoter were constructed. Promoter activity was evaluated by measuring luciferase activity after transfection into PMVECs (Fig. 5a, left). The pGL3-Control vector, containing both SV40 promoter and enhancer sequences, served as a positive control. The full-length CLDN8 putative promoter and the −1,000 to −1 truncation showed stronger activity than the other truncations (*p* < 0.001, *p* < 0.001, respectively; Fig. 5a, right). The activity of these two effective promoter regions was also assessed in PMVECs treated with JXwn06 CM and mock CM, revealing reduced luciferase activity in JXwn06 CM-treated groups, consistent with decreased CLDN8 mRNA expression after treatment (*p* < 0.05, *p* < 0.05, respectively; Fig. 5b).

Next, to screen for potential transcriptional regulators targeting the CLDN8 promoter, purified nuclear proteins from PMVECs treated with JXwn06 CM or mock CM were subjected to DNA pulldown assay followed by mass spectrometry (MS) analysis. Based on the LFQ intensity differences between JXwn06 CM and mock CM-treated groups and their potential roles in transcriptional regulation, the protein interleukin-2 enhancer binding factor 2 (ILF2) was selected for further confirmation (Figs. 5c, S1 and S2) [23]. Overexpression of ILF2 in PMVECs inhibited luciferase activity driven by the CLDN8 promoter sequence (*p* < 0.001; Figs. 5d and S3) and reduced CLDN8 expression levels (*p* < 0.01; Fig. 5e–f). Conversely, ILF2 knockdown by siRNA significantly increased CLDN8 transcription (*p* < 0.001; Fig. 5g and h). The transcription level of ILF2 in PMVECs treated with JXwn06 CM was compared to that in mock CM-treated cells, but no significant difference was observed (*p* > 0.05; Fig. 5i). To further investigate, the intracellular distribution of ILF2 was analyzed using a Nuclear and Cytoplasmic Protein Extraction Kit, showing an increased nuclear to cytoplasmic ratio after JXwn06 CM treatment (Fig. 5j). Consistently, treatment with IL-1β, TNF-α, or JXwn06 CM drove endogenous or exogenous ILF2 into the nucleus from the cytoplasm (Figs. 5k and S4).

### GTF3C2 and THRAP3 contribute to the upregulation of CLDN4 transcription

**Fig. 6:**
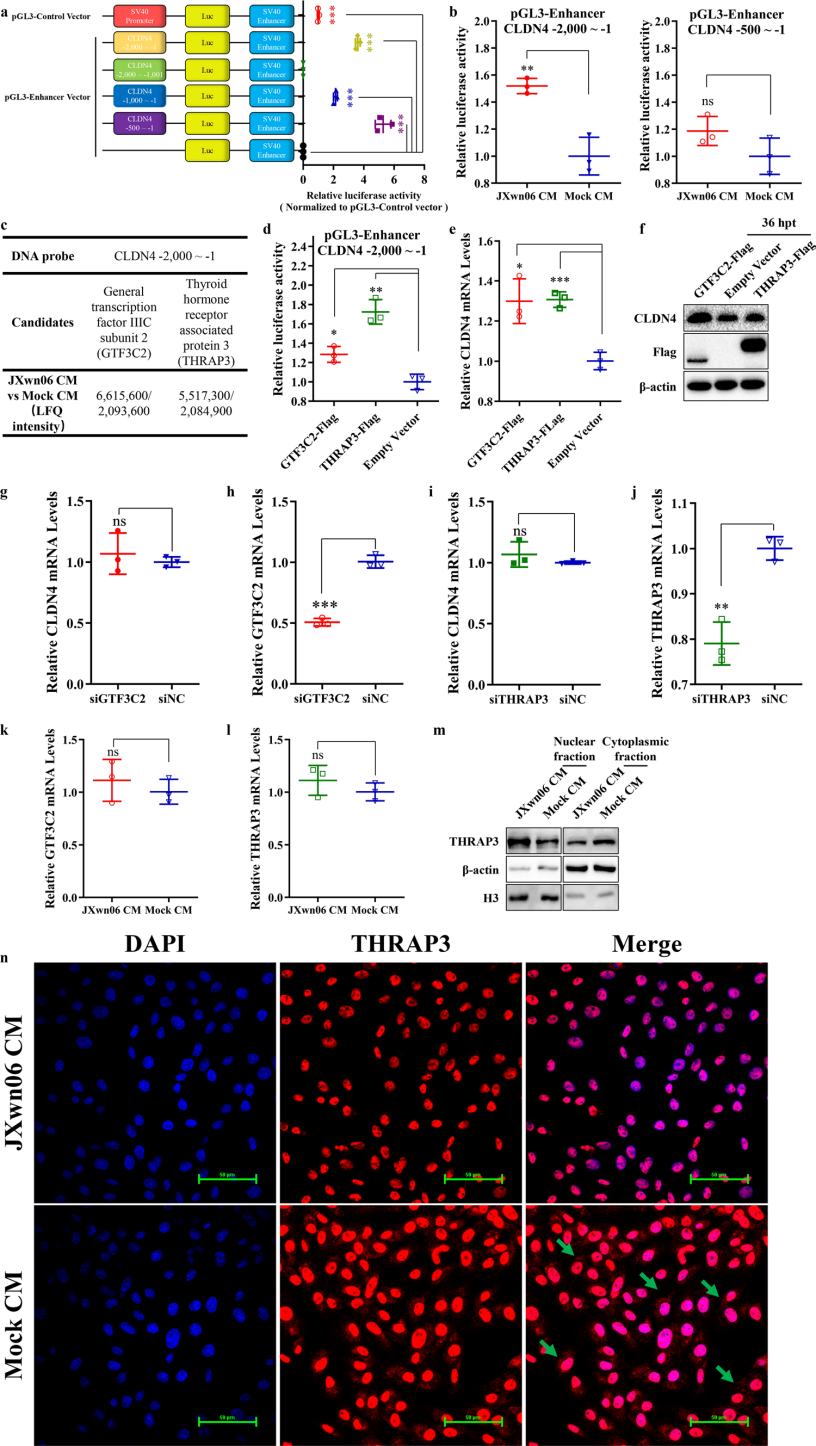
Identify GTF3C2 and THRAP3 that increase CLDN4 transcription by accumulating in PMVECs’ nucleus and targeting the flanking sequence of CLDN4’s promoter. **a** The schematic diagram for construction of the potential promoter regions of CLDN4, and detecting their abilities to initiate the transcription of the reporter gene. **b** The activity of two effective promotor regions of CLDN4 was further evaluated in PMVECs treated with JXwn06 CM or mock CM. **c** GTF3C2 and THRAP3 were enriched in JXwn06 CM treated group, compared with mock CM, as detected by DNA pulldown and MS analysis. **d** Relative luciferase activity in Flag-tagged GTF3C2 (GTF3C2-Flag) or THRAP3-Flag transfected PMVECs. **e** Relative abundance of CLDN4 mRNA in GTF3C2-Flag or THRAP3-Flag transfected PMVECs. The level of CLDN4 mRNA was normalized against β-actin and then compared to the empty vector group. **f** Western blotting analysis of CLDN4 protein level in PMVECs transfected with GTF3C2-Flag or THRAP3-Flag. β-actin served as the loading control. **g**–**j** Relative abundance of CLDN4 mRNA in the PMVECs with GTF3C2 (**g**) or THRAP3 (**i**) knockdown, respectively. The knockdown efficiency of GTF3C2 (**h**) and THRAP3 (**j**) was confirmed by qPCR. **k** and **l** Relative abundance of GTF3C2 and THRAP3 mRNA in JXwn06 CM treated PMVECs. **m** PMVECs were stimulated with JXwn06 or mock CM, at the 6 h post-treatment, and the cell cytoplasmic and nuclear fractions were prepared and assayed for the presence of THRAP3. β-actin and H3 were used as fractionation quality control. **n** The location of endogenous THRAP3 in JXwn06 CM treated PMVECs. Mock CM treated PMVECs served as the negative control. JXwn06 CM facilitates endogenous THRAP3 accumulated in the nucleus (green arrows show endogenous THRAP3 located in the cytoplasm). The fluorescent images were captured with the Nikon A1 confocal microscope (Scale bar, 50 μm). The data are shown as means ± SD (error bars), n = 3 independent experiments were performed in triplicate. Statistical analyses were performed by two-tailed Student’s t-test, and asterisks indicate statistical significance (ns, *p* > 0.05; *, *p* < 0.05; **, *p* < 0.01; ***, *p* < 0.001)

To identify the factors responsible for the transcriptional regulation of CLDN4, a process similar to that used for CLDN8 was employed. The full-length and −500 to −1 truncation of the putative CLDN4 promoter in the pGL3-Enhancer Vector exhibited higher activity than the other truncations (*p* < 0.001, *p* < 0.001, respectively; Fig. 6a), prompting further analysis in PMVECs treated with JXwn06 CM. The relative luciferase activity indicated that the promoter activity of the plasmid containing the full-length putative promoter was upregulated by JXwn06 CM (*p* < 0.01), while the −500 to −1 truncation was not (*p* > 0.05), suggesting that the regulatory region may be located within the −500 to −1 flank (Fig. 6b). Consequently, the full-length putative promoter was used for DNA pulldown assays. Following the screening criteria for CLDN8, the general transcription factor III C subunit 2 (GTF3C2) [24], and the thyroid hormone receptor-associated protein 3 (THRAP3)[25], which were highly enriched from DNA pulldown and mass spectrometry (MS) analysis using a biotin-labeled CLDN4 promoter sequence, were selected for further confirmation (Figs. 6c, S5 and S6).

Firstly, overexpression of GTF3C2 or THRAP3 increased the relative luciferase activity of the pGL3-Enhancer CLDN4 −2,000 to −1 construct (*p* < 0.05, *p* < 0.01, respectively; Fig. 6d), as well as the transcriptional and expression levels of CLDN4 (*p* < 0.05, *p* < 0.001, respectively; Fig. 6e and f). RNAi knockdown of neither GTF3C2 (*p* > 0.05; Fig. 6g–h) nor THRAP3 (*p* > 0.05; Fig. 6i–j) altered the transcriptional level of CLDN4. Similar to the findings with CLDN8, JXwn06 CM did not significantly affect the transcriptional levels of these factors (*p* > 0.05, *p* > 0.05, respectively; Fig. 6k and l) but did increase their nuclear distribution (Fig. 6m). As shown in Fig. 6n and Fig. S7, IL-1β, TNF-α, or JXwn06 CM could induce the endogenous THRAP3 or exogenous GTF3C2 and THRAP3 to translocate from the cytoplasm to the nucleus.

### *miR-185* reduces CLDN8 expression post-transcriptionally

**Fig. 7:**
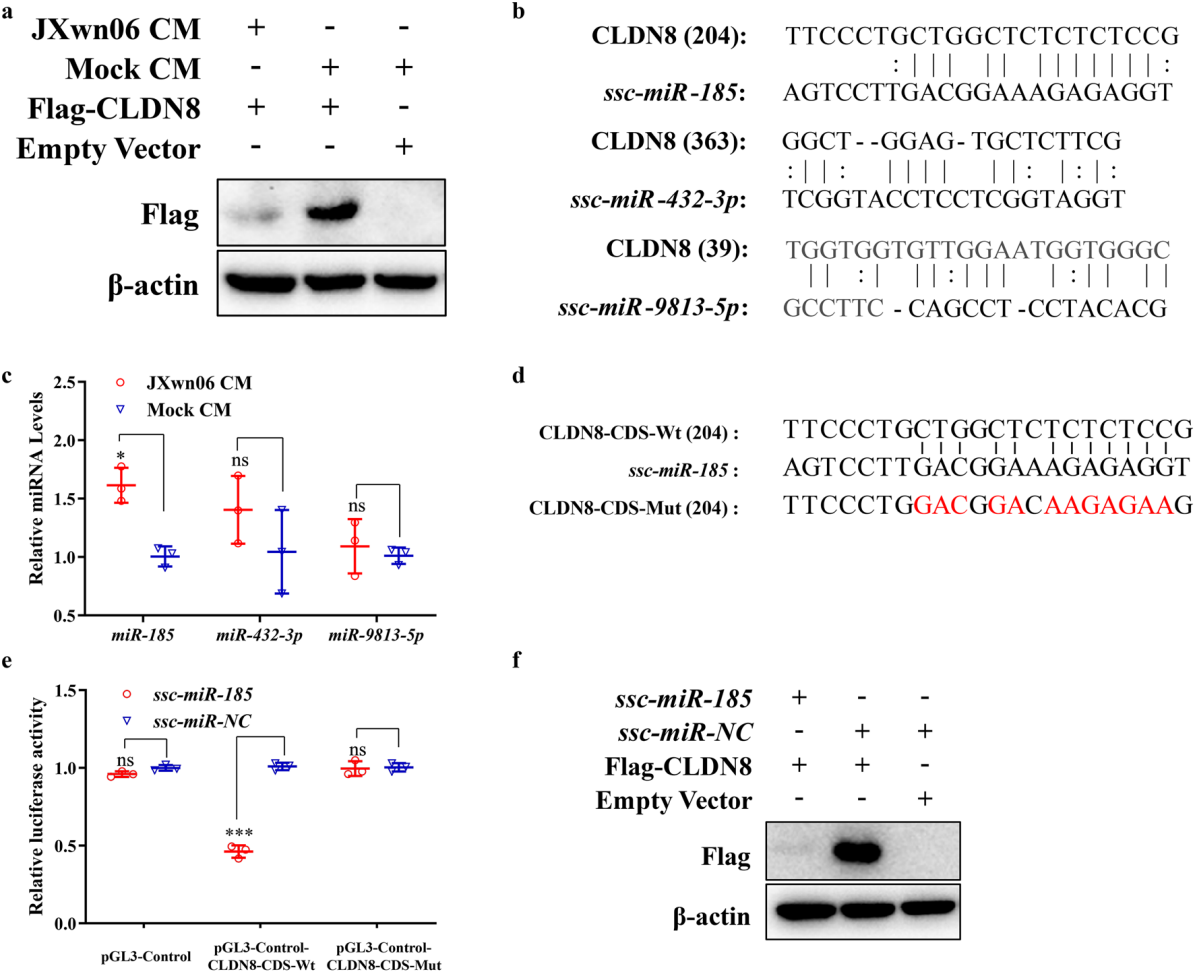
*ssc-miR-185* inhibits CLDN8 expression post-transcriptionally. **a** The expression level of transfected exogenous CLDN8 in PMVECs treated with JXwn06 CM or mock CM. β-actin served as the loading control. **b** Prediction of target sites of *ssc-miR-185*, *ssc-miR-432-3p*, and *ssc-miR-9813-5p* in CLDN8 CDS region. **c** Relative abundance of miRNAs in JXwn06 CM treated PMVECs. The levels of miRNAs were normalized against U6 and then compared to the mock CM treated group. **d** Identification of target sites of *ssc-miR-185* in CLDN8 CDS region. Shown is a diagram of predicted target sites for *ssc-miR-185* in the CLDN8 CDS region. The target sites of the *ssc-miR-185* seed sequence were mutated as indicated in red. **e** Then PMVECs were co-transfected with miRNA mimic and the wild-type (Wt) or mutant (Mut) luciferase reporter plasmid. **f** Western blotting analysis of exogenous CLDN8 protein level in *ssc-miR-185* mimic transfected PMVECs. β-actin served as the loading control. The data are shown as means ± SD (error bars), n = 3 independent experiments were performed in triplicate. Asterisks indicate statistical significance (ns, *p* > 0.05; *, *p* < 0.05; ***, *p* < 0.001)

The studies above have demonstrated that proinflammatory factors IL-1β and TNF-α inhibit the expression of CLDN8 at the transcriptional level by promoting the accumulation of ILF2 in the nucleus. However, it was observed that exogenously expressed CLDN8, driven by the CMV promoter, could also be inhibited by treatment with JXwn06 CM (Fig. 7a, compare lane 1 with 2), suggesting that CLDN8 might be regulated post-transcriptionally as well. Given that microRNAs (miRNAs), small noncoding RNAs of 21–22 nucleotides in length, are essential for regulating gene expression post-transcriptionally [26, 27], three miRNA target prediction algorithms—miRanda, PITA, and RNAhybrid—were employed to predict potential miRNAs targeting the CLDN8 mRNA sequence. Among 244 candidate miRNAs, *ssc-miR-185*, *ssc-miR-432-3p*, and *ssc-miR-9813-5p* were consistently identified by all three algorithms. The miRNA target sites on the CLDN8 mRNA were further identified using the RNA22.v2 database (Fig. 7b).

The transcription levels of *ssc-miR-185*, *ssc-miR-432-3p*, and *ssc-miR-9813-5p* in PMVECs treated with JXwn06 CM were assessed using the miRcute Plus miRNA qPCR Kit, revealing a significant upregulation of *ssc-miR-185* after treatment (*p* < 0.05; Fig. 7c). To further validate the regulatory effect of *ssc-miR-185* on CLDN8, the wild-type CDS region of CLDN8 (CLDN8-CDS-Wt) and a mutated sequence (CLDN8-CDS-Mut) were cloned downstream of the *luc* + gene in the pGL3-Control vector using the *Xba* I restriction site. The recombinant reporter plasmids were then co-transfected with an miRNA mimic of *ssc-miR-185* into PMVECs (Fig. 7d). Compared to the negative control (NC) group, the luciferase activity of the plasmid containing CLDN8-CDS-Wt was significantly reduced upon transfection with the *ssc-miR-185* mimic, while the inhibitory effect was abolished when the *ssc-miR-185* binding sites were mutated (*p* < 0.001; Fig. 7e). Similarly, transfection with the *ssc-miR-185* mimic significantly reduced the exogenous protein expression level of CLDN8 (Fig. 7f, compare lane 1 with 2). These findings indicate that JXwn06 CM treatment can also reduce CLDN8 expression post-transcriptionally by upregulating *ssc-miR-185*, which targets the CDS of CLDN8.

## Discussion

In the acute phase of acute lung injury (ALI), activated resident alveolar macrophages release various potent proinflammatory mediators. Early viral replication creates an inflammatory niche by promoting the production of cytokines and chemokines, which are closely associated with the severity of pathology and virus proliferation [5, 28, 29]. To more accurately mimic the in vivo lung environment, alveolar lavage fluid (ALF) was collected from HP-PRRSV-infected pigs at 4 days post-inoculation (dpi), a time when fever and other clinical symptoms were first observed, and when microscopic pathological changes indicative of endothelial barrier leakage were evident. Additionally, isolated primary PMVECs were also utilized to confirm changes in endothelial permeability and TJs expression following treatment with JXwn06 CM (Fig. S8). During PRRSV infection, activated alveolar macrophages and other inflammatory cells secrete proinflammatory cytokines such as TNF-α, IL-1α/β, IL-6, IL-8, and IL-12, as well as TGF-β, to recruit neutrophils and lymphocytes to the site of infection and facilitate pathogen clearance [30, 31]. Therefore, IL-1, IL-6, TNF-α, and TGF-β were selected for further testing based on previous studies highlighting their potential roles in disrupting the endothelial barrier [32–35], The direct stimulation function of PRRSV viral particles was also ruled out in this study. IL-1 and TNF-α, which were highly induced in both the supernatant of JXwn06-infected PAMs and the alveolar lavage fluid (ALF) of infected pigs, were identified as significantly increasing paracellular permeability. Although the effects of all cytokines in the supernatants and ALF could not be evaluated, the experiments involving signal pathway blocking provided solid evidence to support this point. Receptor antagonists or antibodies targeting these two cytokines (Anakinra and Adalimumab) were able to almost fully block the effect of JXwn06 CM on endothelial permeability. These findings offer valuable insights for developing therapeutic methods and strategies for anti-PRRSV breeding. Unfortunately, the high cost of commercial neutralizing antibodies and the extensive amount required for pig studies make it impractical to evaluate their therapeutic effects in vivo. However, numerous studies have documented that TNF-α and IL-1β are abundant in the lungs or serum of PRRSV-infected pigs, with highly pathogenic PRRSV strains producing higher and earlier levels of TNF-α and IL-1β [4, 36].

The vascular endothelium consists of a monolayer of tightly assembled endothelial cells, and thus, the integrity of the endothelial barrier is crucial for its permeability. The three primary causes of a compromised endothelial barrier are cell junctions, apoptosis, and cytoskeletal remodeling [10, 37]. Initially, the apoptosis of PMVECs was assessed using an Annexin V/Propidium Iodide staining assay following treatment with JXwn06 CM, IL-1β, or TNF-α. The results indicated that apoptosis was minimally induced when significant changes in permeability and the expression levels of CLDN8 and CLDN4 were observed. Given that dynamic cytoskeletal remodeling also plays a role in regulating junction assembly and function [38], the complexity of the system has constrained our focus to the regulation of TJ proteins in this study.

Claudins, the major components of tight junctions (TJs), are categorized into barrier-forming and channel-/pore-forming proteins, creating a complex network to control intercellular permeability [12, 13]. Due to their tissue-specific expression and complex interaction patterns, different claudins play distinct roles in regulating fluid diffusion between cells (paracellular flux) [39]. In our previous study, CLDN8 and CLDN4 in PMVECs were found to be dysregulated when the cells interacted with HP-PRRSV-infected PAMs [6]. Previous studies have shown that CLDN4 forms a paracellular chloride channel in collecting duct cells, requiring CLDN8 for TJ assembly in the epithelium, and increased CLDN4 levels are associated with improved lung water clearance and reduced damage to the physiological lung barrier [15, 40, 41]. Conversely, overexpression of CLDN8 selectively decreases the permeability of cations through TJs, specifically Na + , K + , H + , and ammonium [16]. To further investigate their roles in PMVEC permeability changes, CLDN8 overexpression and/or CLDN4 RNAi knockdown were performed to assess their antagonistic effects on the permeability increase induced by JXwn06 CM. Interestingly, CLDN8 overexpression can almost fully restore the TEER in JXwn06 CM-treated PMVECs, but it requires the additional knockdown of CLDN4 to block the increased permeability caused by JXwn06 CM. Moreover, individual CLDN4 knockdown can partially increase TEER and reduce permeability in JXwn06 CM-treated PMVECs, a function that appears to differ from its role in epithelial cells. In another study on the development of acute respiratory distress syndrome (ARDS) promoted by IL-1β, CLDN4 was found to be upregulated in lung epithelial cells, along with the downregulation of CLDN18, which the authors interpreted as a compensatory expression to impair lung barrier function in ARDS [42]. Indeed, overexpression of CLDN4 in PMVECs has been shown to increase FITC-Dextran permeability (Fig. 4d–f). Therefore, the ion selectivity or other characteristics of these two TJ proteins during hyperpermeability, as well as the development of potential targeted drugs for ALI treatment, require further exploration.

The transcriptional regulation of TJ genes is a complex process involving numerous signaling pathways, including protein kinase C (PKC), protein kinase A (PKA), protein kinase G (PKG), Rho, mitogen-activated protein kinase (MAPK), phosphatidylinositol 3-kinase (PI3K)/Akt, and Wnt/β-catenin pathways [10]. TNF-α, through the MAPK axis (ERK1/2 and p38), can regulate TJ expression by interacting with nuclear proteins and transcription factors such as NF-κB, Snail1, KLF2, and p53, and it can crosstalk with other signals like VEGF, TLR, MLC2, STAT3, and SGK1 [10]. IL-1β has also been reported to promote the development of acute respiratory distress syndrome (ARDS) by regulating CLDN18 through the human epidermal growth factor receptor (HER) pathway (IL-1β-HER2/HER3 axis) [42]. However, the factors involved in regulating the expression of CLDN8 and CLDN4 in PMVECs treated with stimulators are not well understood. To address this, the promoter regions of these genes were cloned and utilized to identify regulatory factors within the cellular nucleus.

ILF2, which encodes NF45, was originally identified as a regulatory subunit of the NF90/NF110 complexes that bind to the antigen recognition response element in the interleukin-2 promoter. It is involved in mitotic control and various aspects of RNA metabolism, including transcription, RNA transport, mRNA stability, and translation [43]. The general transcription factor III (GTF3) family, consisting of GTF3A, GTF3B, GTF3C1, and GTF3C2, functions as RNA polymerase III transcription factors, inducing the transcription of 5S ribosomal (r)RNA genes involved in ribosomal large-subunit biogenesis [44]. Thyroid hormone receptor-associated protein 3 (THRAP3) is a multifunctional protein that acts as a transcription coactivator for the circadian clock factor CLOCK-BMAL1 [45], and as a corepressor by inhibiting the transcription factor SOX9 during chondrogenesis [46]. It also has RNA-binding activity and is essential for RNA splicing and the export of transcripts involved in the cellular DNA damage response [47, 48]. These three factors were initially identified from promoter binding proteins in mass spectrometry (MS) analysis due to their potential role in regulating gene transcription. Subsequent analysis of their transcriptional regulation effects, expression levels, and distribution in PMVECs during stimulation revealed that JXwn06 CM treatment did not significantly alter the expression levels of these factors but increased their nuclear distribution, where they can either inhibit or activate the transcription of CLDN8 and CLDN4. Efforts to further delineate the interaction site of ILF2 with the CLDN8 promoter through luciferase experiments with truncated promoters indicated a region of interaction roughly between -300 and -1. However, identifying more precise sites was challenging due to the potential loss of promoter activity with further truncation. In addition to the interactions between these transcription factors and CLDN promoters, the pathways by which TNF-α and IL-1β drive these factors into the nucleus warrant further investigation.

MicroRNAs (miRNAs) have been reported to regulate tight junction proteins, thereby modulating epithelial and endothelial barrier function. For instance, ZO-1, CLDN5, and occludin are positively regulated by *miR-126*, *miR-107*, and *miR-21*, while they are negatively regulated by *miR-181a*, *miR-98*, and *miR-150* [17]. Additionally, we observed that the exogenously expressed CLDN8, driven by the CMV promoter, can be suppressed in PMVECs treated with JXwn06 CM. This led us to speculate that there is post-transcriptional regulation of CLDN8 expression. Further investigation revealed that the upregulated *ssc-miR-185* can reduce CLDN8 expression post-transcription by binding to its protein-coding sequences (CDS).

**Fig. 8:**
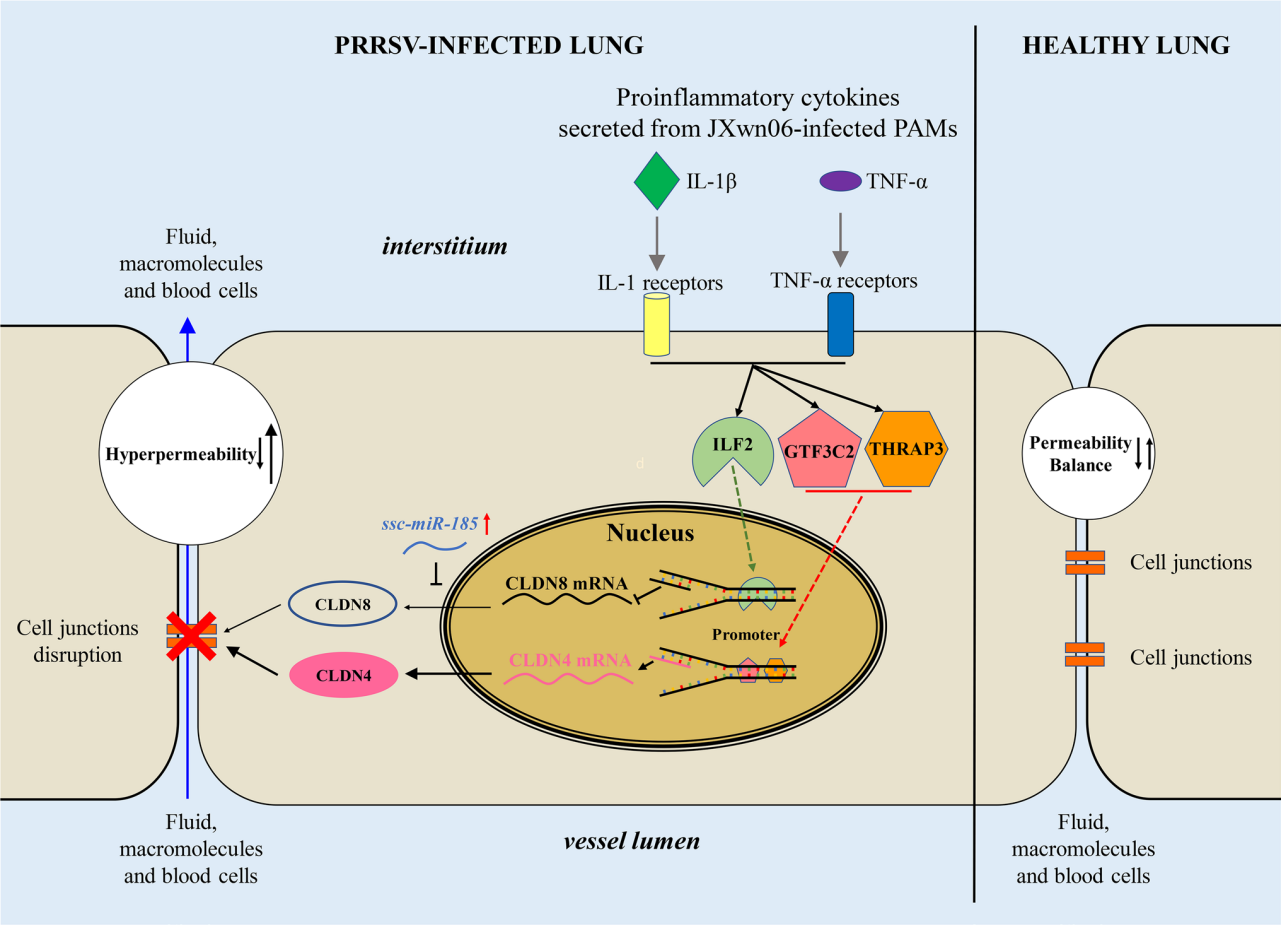
A proposed model of IL-1β and TNF-α enhancing the pulmonary vascular permeability through CLDN8 and CLDN4. Proinflammatory cytokines IL-1β and TNF-α secreted by HP-PRRSV JXwn06-infected primary PAMs trigger pulmonary microvascular endothelial barrier hyperpermeability via both downregulated CLDN8 and upregulated CLDN4. The transcriptional regulator ILF2 translocate into the nucleus in response to proinflammatory cytokine signaling to downregulate the transcription activity of the CLDN8 promoter. In parallel, GTF3C2 and THRAP3 also translocate into the nucleus to upregulate the transcription activity of the CLDN4 promoter. The upregulated *ssc-miR-185* can also inhibit CLDN8 expression post-transcriptionally. All these biological processes result in pulmonary endothelial hyperpermeability

In summary, using an animal inoculation model and an in vitro endothelial cell Transwell culture system, we discovered that IL-1β and TNF-α, released from HP-PRRSV-infected PAMs, can compromise the integrity of the pulmonary vascular barrier by dysregulating the tight junction proteins CLDN8 and CLDN4. This dysregulation is facilitated by the transcription factors ILF2, GTF3C2, and THRAP3, which accumulate in the nucleus of PMVECs to regulate the transcription of CLDN8 and CLDN4. Additionally, the upregulation of *ssc-miR-185* contributes to the suppression of CLDN8 expression by binding to its coding region (Fig. 8). These findings offer novel insights into the function of tight junctions and their regulatory pathways in vascular homeostasis. They provide valuable clues for understanding the mechanisms of acute lung injury (ALI) caused by viral infections and for developing therapeutic strategies that target endothelial barrier function to treat respiratory diseases.

### Supplementary Information

Below is the link to the electronic supplementary material.

Supplementary file1 (DOCX 15 KB)

Supplementary file2 (TIF 10495 KB)

Supplementary file3 (TIF 268 KB)

Supplementary file4 (TIF 632 KB)

Supplementary file5 (TIF 6650 KB)

Supplementary file6 (TIF 13467 KB)

Supplementary file7 (TIF 416 KB)

## Data Availability

All data generated or analyzed during this study are included in this published article and its supplementary information files.
