# Supplementary material for: Disruption of pulmonary microvascular endothelial barrier by dysregulated claudin-8 and claudin-4: uncovered mechanisms in porcine reproductive and respiratory syndrome virus infection
Source: Cell Mol Life Sci. 2024 May 28;81(1):240. doi: 10.1007/s00018-024-05282-4 (PMC11133251; doi:10.1007/s00018-024-05282-4)
Supplement: Supplementary file 1 — Supplementary file1 (DOCX 15 KB) [file 18_2024_5282_MOESM1_ESM.docx]

**Supplementary figure legends**

**Fig. S1 Map of transcription factor candidates of CLDN8 promoter.** The interaction network analysis between total 132 candidates derived from mass spectrometry was conducted by STRING (Version: 12.0, https://cn.string-db.org/).

**Fig. S2 Selection of transcription factor candidates for CLDN8.** ILF2, H1-3, and PARP1, resulting from map of transcription factor candidates of CLDN8 promoter, were transfected into PMVECs, then their effects on CLDN8 mRNA level were measured by real time PCR. The data are shown as means ± SD (error bars), n=3 independent experiments were performed in triplicate. Asterisks indicate statistical significance (*, *p* < 0.05; **, *p* < 0.01).

**Fig. S3 Identification of ILF2’s binding sites region on CLDN8 promoter.** ILF2 or empty vector was co-transfected with different CLDN8 promoter truncations, -500 ~ -1, -500 ~ -301, -300 ~ -1, -500 ~ -101, or -100 ~ -1, into PMVECs, then the transactivation was measured by dual-luciferase reporter system. The data are shown as means ± SD (error bars), n=3 independent experiments were performed in triplicate. Asterisks indicate statistical significance (ns, *p* > 0.05; ***, *p* < 0.001).

**Fig. S4 Subcellular location of exogenous expressed ILF2 in PMVECs.** ILF2 mRNA was cloned into pCAGGS-HA, which tagged with Flag, then the ILF2 or empty vector was transfected into PMVECs, respectively. At ~18 hours post transfection, PMVECs were treated with JXwn06 or mock CM for another 12 h, then fluorescent images were captured with the Nikon A1 confocal microscope (Scale bar, 10 μm).

**Fig. S5 Map of transcription factor candidates of CLDN4 promoter.** The interaction network analysis among total 232 candidates derived from mass spectrometry was conducted by STRING (Version: 12.0, <https://cn.string-db.org/>).

**Fig. S6 Selection of transcription factor candidates for CLDN4.** GTF3C2, THRAP3 and NFIB, resulting from map of transcription factor candidates of CLDN4 promoter, were transfected into PMVECs, then their effects on CLDN4 mRNA level were measured by real time PCR. The data are shown as means ± SD (error bars), n=3 independent experiments were performed in triplicate. Asterisks indicate statistical significance (ns, *p* > 0.05; **, *p* < 0.01).

**Fig. S7 Subcellular location of exogenous expressed GTF3C2 or THRAP3 in PMVECs.** The mRNA of GTF3C2, or THRAP3, was cloned into pCAGGS-HA, which tagged with Flag, then the GTF3C2, or THRAP3 or empty vector was transfected into PMVECs, respectively. At ~18 hours post transfection, PMVECs were treated with JXwn06 or mock CM for another 12 h, then fluorescent images were captured with the Nikon A1 confocal microscope (Scale bar, 10 μm).

**Fig. S8 Tight junction proteins CLDN8 and CLDN4 in primary PMVECs are dysregulated by JXwn06 CM.** The purified primary porcine PMVECs were cultured in RPMI-1640 medium supplemented with 5% FBS at 37 °C under a humid 5% CO_2_ atmosphere. When the cells became confluent, JXwn06 CM or mock CM was added to the culture medium for another 12 or 24 hours. (**A**) Relative abundance of CLDN8 and CLDN4 mRNA in primary PMVECs treated with JXwn06 CM at 12 hpt. The level of target gene mRNA was normalized against β-actin and then compared to the mock CM-treated group. (**B**) Western Blotting analysis of CLDN8 and CLDN4 protein levels in PMVECs treated with JXwn06 CM at 12 and 24 hpt. β-actin served as the loading control. The data are shown as means ± SD (error bars), n=3 independent experiments were performed in triplicate. Asterisks indicate statistical significance (**, *p* < 0.01; ***, *p* < 0.001).
