# Supplementary figures and images for: Disruption of pulmonary microvascular endothelial barrier by dysregulated claudin-8 and claudin-4: uncovered mechanisms in porcine reproductive and respiratory syndrome virus infection
Source: Cell Mol Life Sci. 2024 May 28;81(1):240. doi: 10.1007/s00018-024-05282-4 (PMC11133251; doi:10.1007/s00018-024-05282-4)

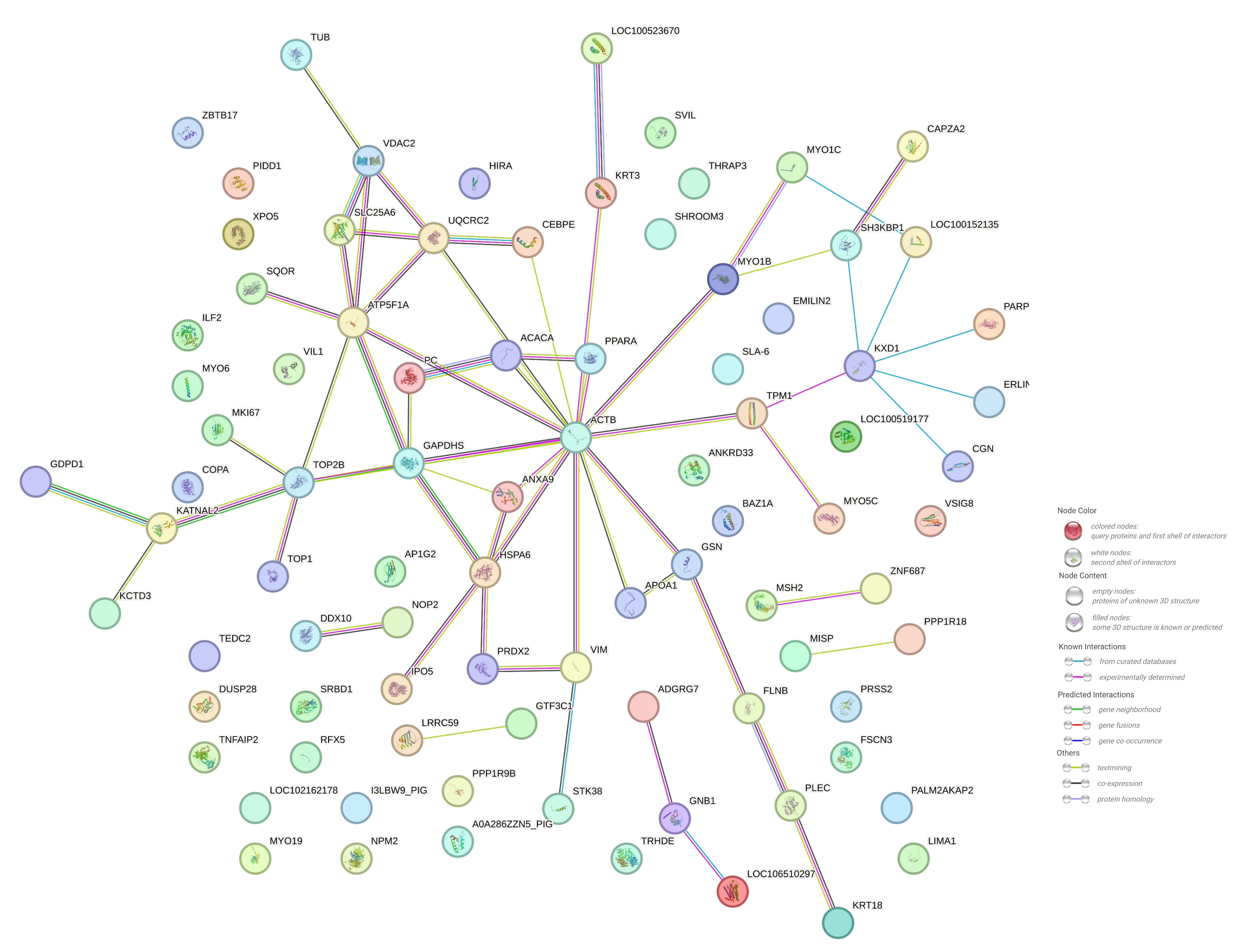

Supplement: Supplementary file 2 — Supplementary file2 (TIF 10495 KB) [file 18_2024_5282_MOESM2_ESM.tif]

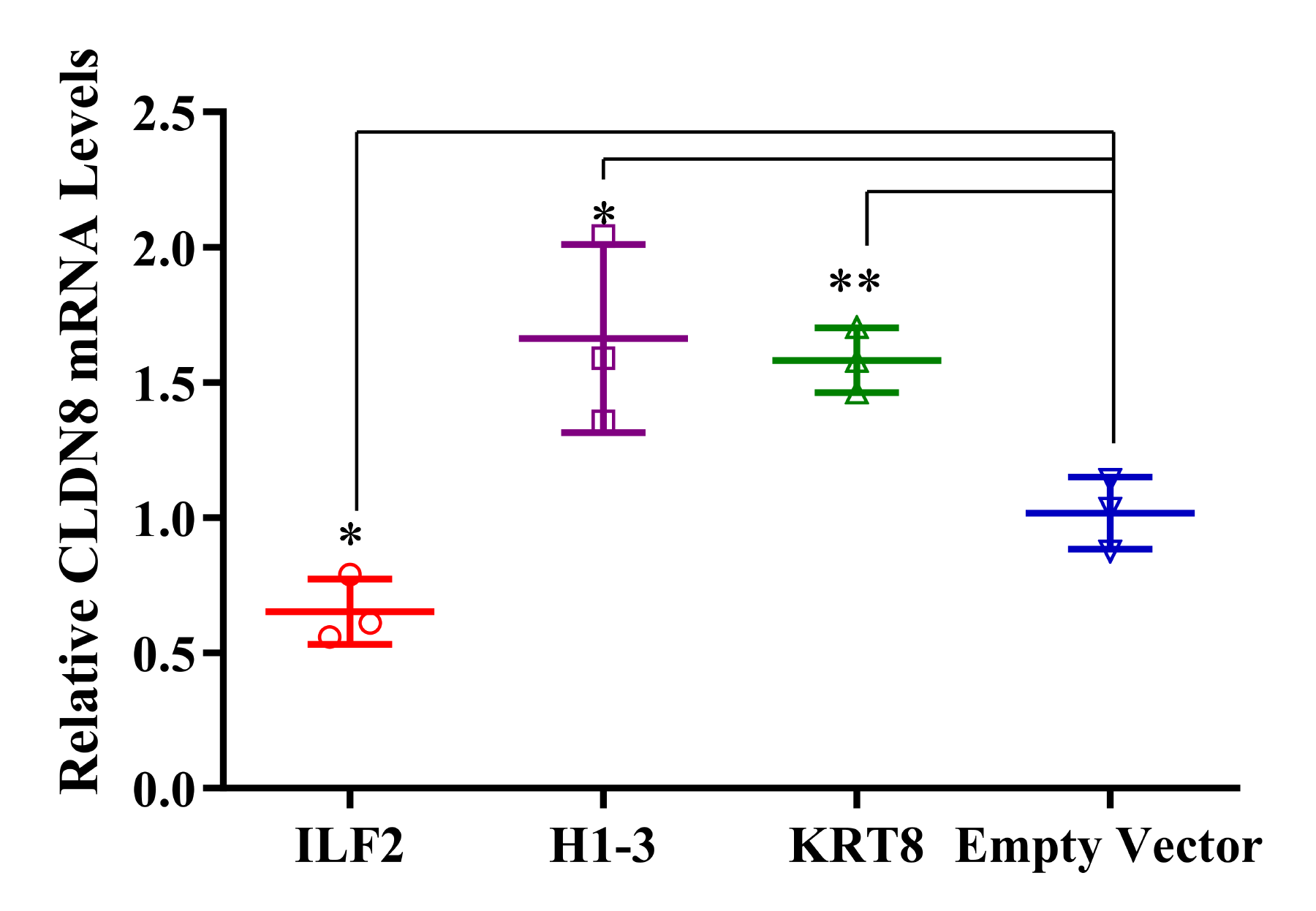

Supplement: Supplementary file 3 — Supplementary file3 (TIF 268 KB) [file 18_2024_5282_MOESM3_ESM.tif]

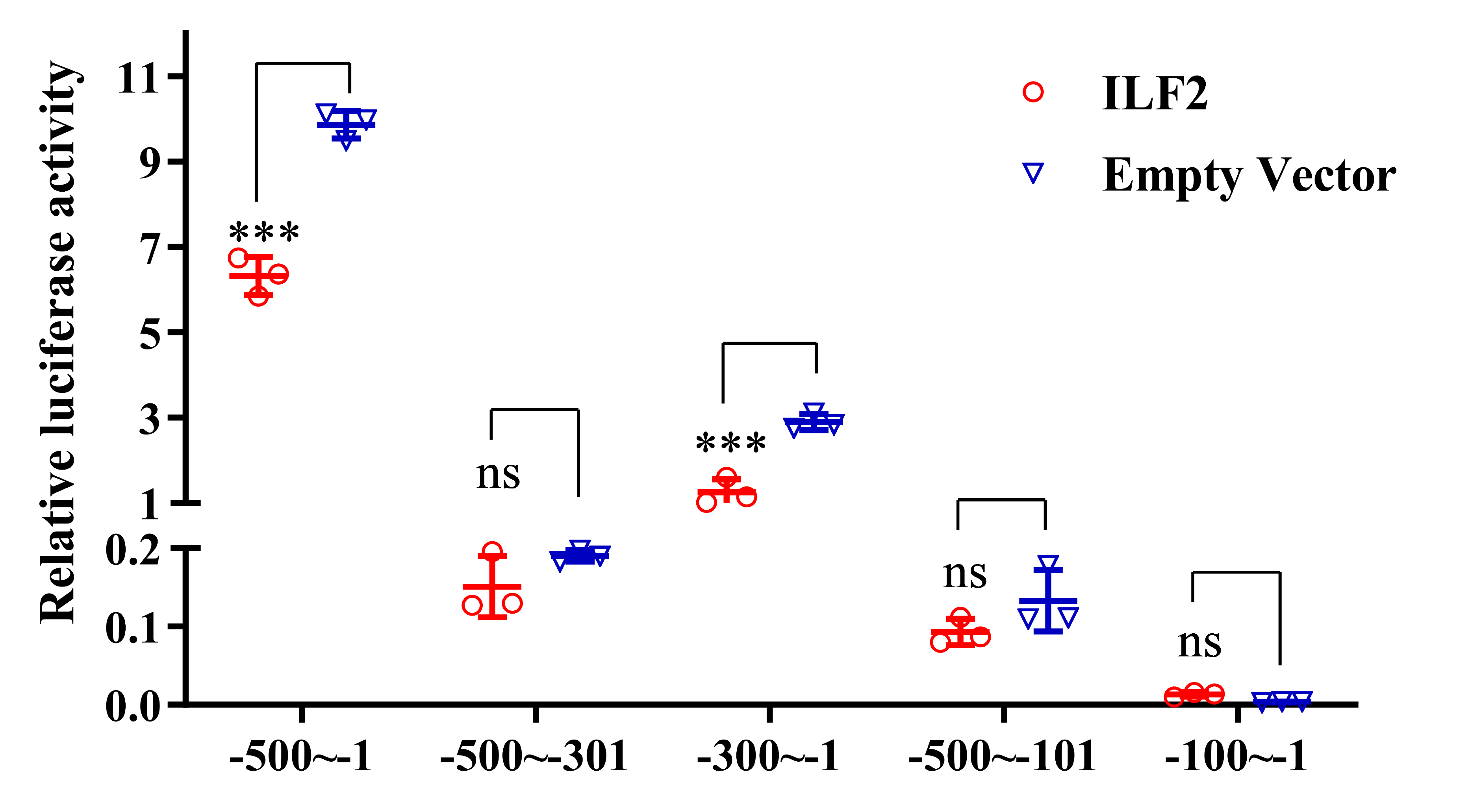

Supplement: Supplementary file 4 — Supplementary file4 (TIF 632 KB) [file 18_2024_5282_MOESM4_ESM.tif]

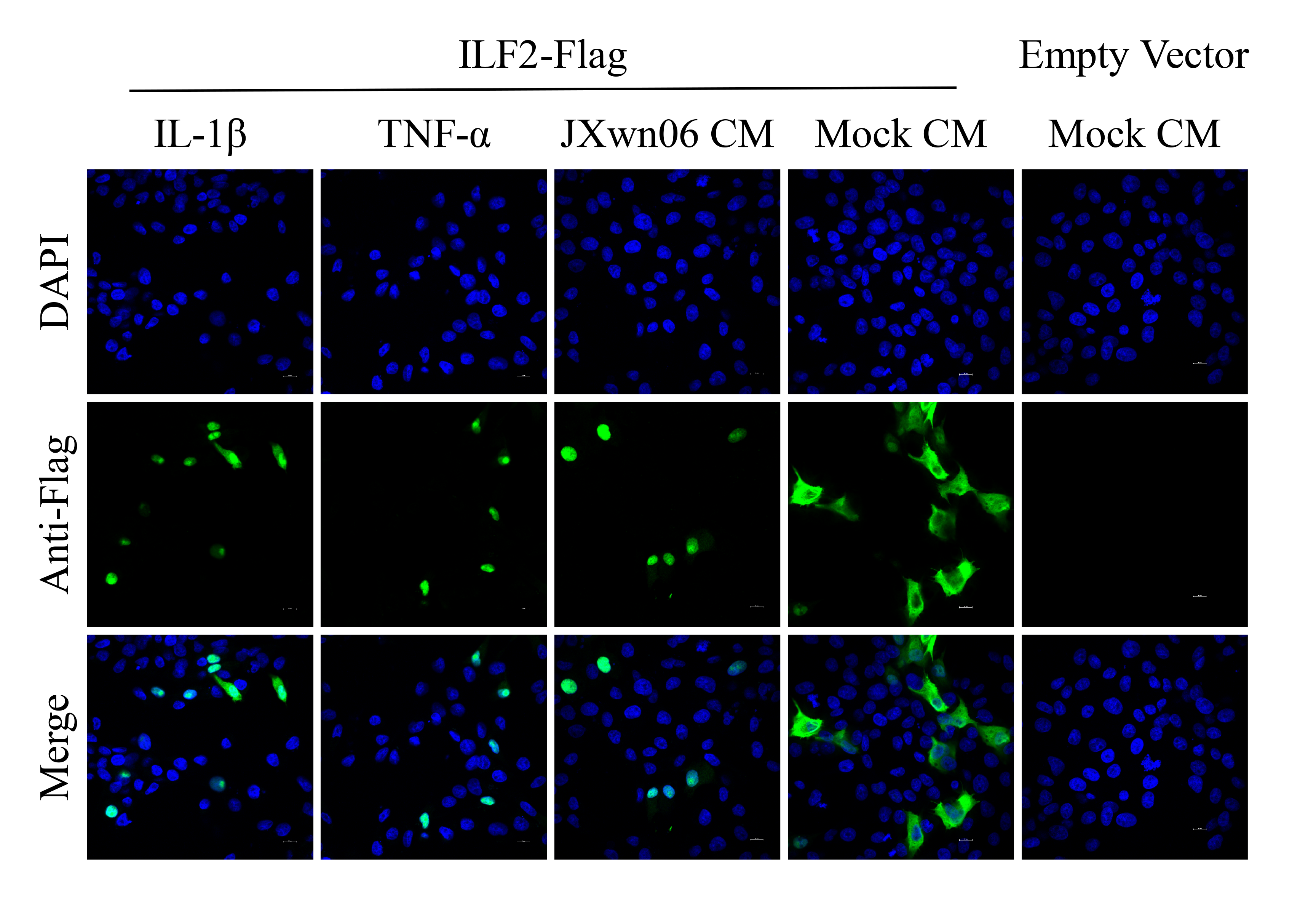

Supplement: Supplementary file 5 — Supplementary file5 (TIF 6650 KB) [file 18_2024_5282_MOESM5_ESM.tif]

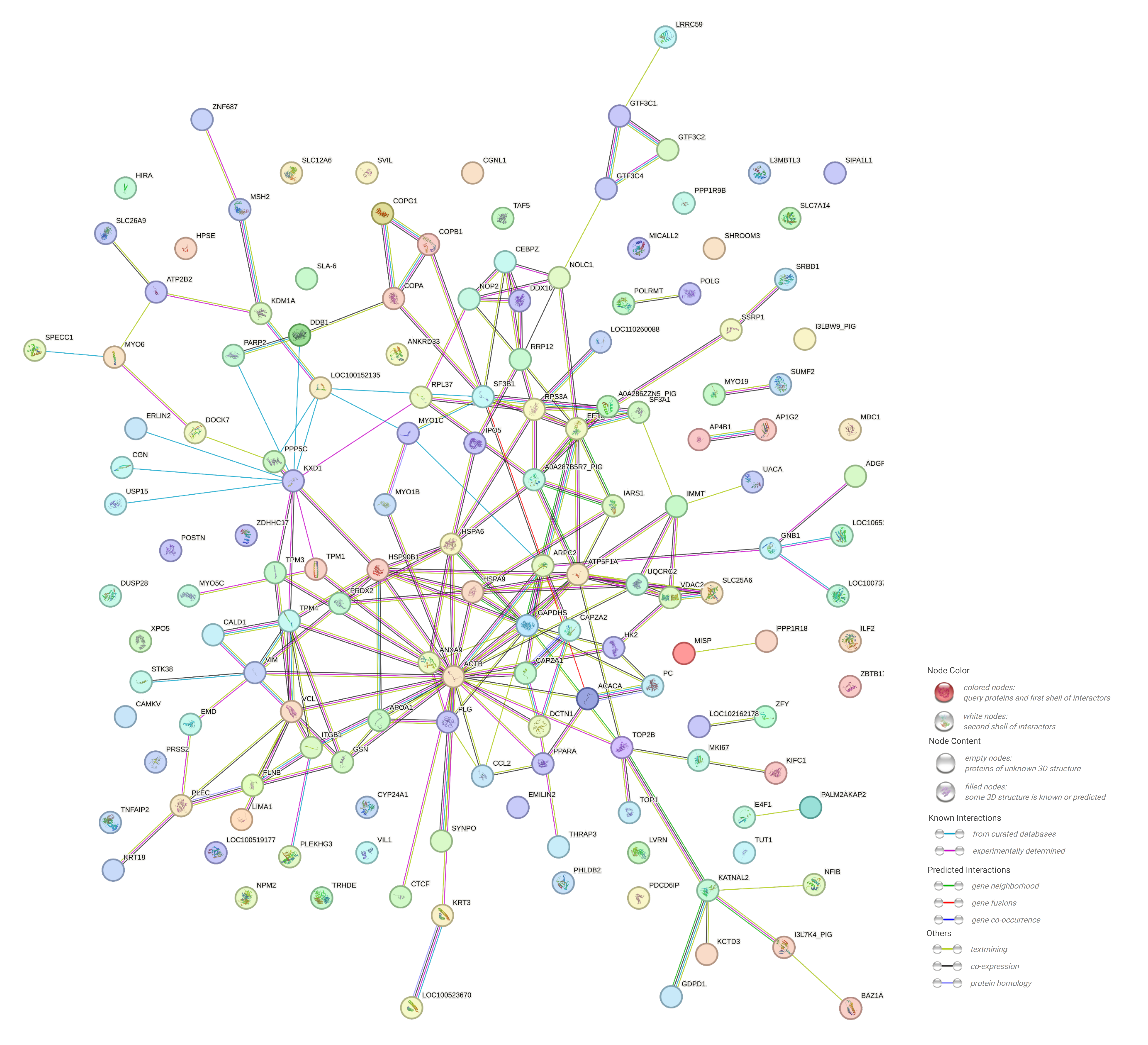

Supplement: Supplementary file 6 — Supplementary file6 (TIF 13467 KB) [file 18_2024_5282_MOESM6_ESM.tif]

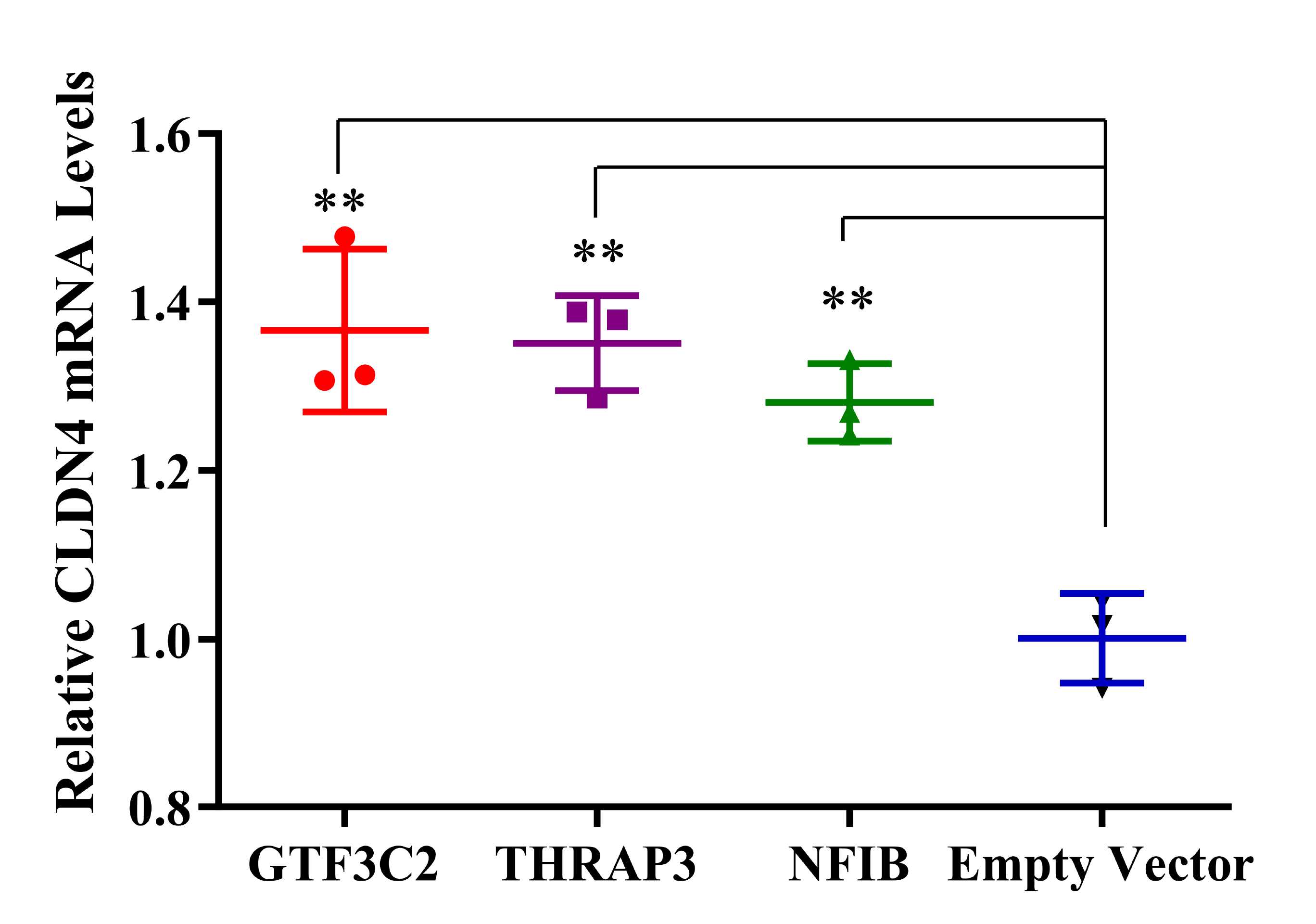

Supplement: Supplementary file 7 — Supplementary file7 (TIF 416 KB) [file 18_2024_5282_MOESM7_ESM.tif]
